# Hydrogel Elastic Energy: A Stressor Triggering an Adaptive Stress‐Mediated Cell Response

**DOI:** 10.1002/adhm.202402400

**Published:** 2024-11-13

**Authors:** Sara Lipari, Pasquale Sacco, Michela Cok, Francesca Scognamiglio, Maurizio Romano, Francesco Brun, Piero Giulio Giulianini, Eleonora Marsich, Finn L. Aachmann, Ivan Donati

**Affiliations:** ^1^ Department of Life Sciences University of Trieste Via L. Giorgieri 5 Trieste 34127 Italy; ^2^ Department of Engineering and Architecture University of Trieste Via A. Valerio 6/1 Trieste 34127 Italy; ^3^ Department of Medicine Surgery and Health Sciences University of Trieste Piazza dell'Ospitale 1 Trieste 34129 Italy; ^4^ Department of Biotechnology and Food Science Norwegian Biopolymer Laboratory (NOBIPOL) NTNU Norwegian University of Science and Technology Sem Sælands vei 6/8 Trondheim 7491 Norway

**Keywords:** cellular stress state, extracellular matrix mimic, hydrogel, mechanical feedback, mechanosensing

## Abstract

The crosstalk between the cells and the extracellular matrix (ECM) is bidirectional and consists of a pushing/pulling stretch exerted by the cells and a mechanical resistance counteracted by the surrounding microenvironment. It is widely recognized that the stiffness of the ECM, its viscoelasticity, and its overall deformation are the most important traits influencing the response of the cells. Here these three parameters are combined into a concept of elastic energy, which in biological terms represents the mechanical feedback that cells perceive when the ECM is deformed. It is shown that elastic energy is a stress factor that influences the response of cells in three‐dimensional (3D) cultures. Strikingly, the higher the elastic energy of the matrix and thus the mechanical feedback, the higher the stress state of the cells, which correlates with the formation of G3BP‐mediated stress granules. This condition is associated with an increase in alkaline phosphatase (ALP) activity but a decrease in gene expression and is mediated by the nuclear translocation of Yes‐associated protein (YAP). This work supports the importance of considering the elastic energy as mechano‐controller in regulating cellular stress state in 3D cultures.

## Graphical abstract

In three‐dimensional (3D) microenvironments, cells deform the surrounding extracellular matrix and experience mechanical feedback that influences their response. The resulting cellular stress state promotes the formation of stress granules, increased alkaline phosphatase activity, and nuclear translocation of Yes‐associated protein. The mechanical feedback, proposed here as elastic energy, can be considered as a mechano‐controller in regulating cellular response in 3D cultures.

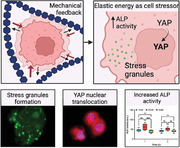

## 1 Introduction

In natural tissues, cells live in three‐dimensional (3D) microenvironments where they exert traction forces or alter their volume, thereby deforming the surrounding extracellular matrix (ECM) and influencing its physical composition.^[^ ^1^, ^2^, ^3^, ^4^, ^5^ ^]^ This physical interaction plays a significant role in biological and pathological processes, including cancer development.^[^ ^4^, ^6^, ^7^, ^8^, ^9^, ^10^ ^]^ While studies using two‐dimensional (2D) culture models have convincingly demonstrated the impact of mechanical cues like stiffness and viscoelasticity on cellular processes such as cell migration, proliferation, malignancy, and differentiation,^[^ ^8^, ^11^, ^12^ ^]^ transitioning to 3D culture systems provides a more physiologically relevant milieu that closely mimics in vivo conditions.

A remarkable mechanosensing process of cells in 3D environments entails the regulation of their cell volume in response to the mechanics of the network, which is particularly evident when cells are mechanically confined without integrin‐binding ligands.^[^ ^13^, ^14^, ^15^ ^]^ Key to this regulation are mechanosensitive ion channels, notably TRPV4 or Piezo1 channels, serving as central sensors for the dynamic adjustment of cell volume in response to external mechanical signals.^[^ ^16^, ^17^ ^]^ Consequently, cells can react to these stimuli by initiating various intracellular processes ranging from deposition of cartilage matrix to differentiation of stem cells for osteogenesis and proliferation of cancer cells.^[^ ^17^, ^18^, ^19^, ^20^, ^21^ ^]^

Nevertheless, studies on the mechanosensing of cells in connection with the expansion of cell volume are still in their infancy. The concept that cells expand their volume to sense the surrounding microenvironment is closely related to the elastic resistance of the network. Mechanically, this means that the higher the elasticity of the network, the higher the resistance to the expansion of the cell volume. From a biological point of view, this condition can be viewed as a vicious loop between cell volume expansion and the mechanical feedback that establishes a mechanoreciprocity sensing. It has recently been demonstrated that there is an elastic energy sensing that significantly affects cell mechanotransmission and transduction on viscoelastic substrates.^[^ ^22^ ^]^ Considering all the above, this last finding prompts us to ask whether cells in a 3D environment perceive this mechanical feedback.

Here, we prepared viscoelastic ECM mimics using an alginate methacrylate (ALMA) with tunable mechanics and suitable for 3D cell culture to investigate the influence of mechanical elastic energy feedback on cell behavior. This type of energy is found to be a cell stressor affecting gene expression, enzymatic activity, and translocation of the mechanosensor Yes‐associated protein (YAP).

## 2 Results

### 2.1 ALMA and Unmodified Alginate Share Similar Macromolecular Properties

In the synthesis of ALMA, the hydroxyl groups of alginate reacted with methacrylic anhydride at basic pH.^[^ ^23^ ^]^ To confirm the coupling of the methacrylate moiety to alginate, we inserted it to a well‐characterized G oligomer (DP 26) and analyzed the product by NMR spectroscopy. All the chemical shifts were assigned and are listed in Figure S1 (Supporting Information). Both HMBC and NOESY spectra were scrutinized to identify a connection between the methacrylate moiety and the G‐oligomer, but no correlation was found. However, diffusion‐ordered spectroscopy (DOSY) was applied, where signals from the G‐oligomer and methacrylate moiety exhibited the same diffusion, indirectly confirming that their covalent linkage. ^1^H‐NMR spectroscopy allowed evaluating of the effects of various factors on the degree of substitution, such as polymer concentration, NaOH concentration, amount of methacrylic anhydride, reaction time, and temperature (Table S1 and Figure S2, Supporting Information). The methacrylate insertion onto the alginate backbone showed a non‐monotonic trend in relation to both the reaction temperature, with the highest degree of substitution observed at 25 °C, and the amount of methacrylic anhydride. In the latter case, an R_M.An_ of 10.6 led to the highest degree of substitution while a competing hydrolysis of the anhydride by OH^−^ ions became relevant for higher R_M.An._ values (Figure S2 and Appendix SA1, Supporting Information). While the degree of substitution increased slightly with longer reaction times, the increase in the polymer concentration, and thus in the viscosity of the solution,^[^ ^24^ ^]^ showed the opposite trend.

**Figure 1:**
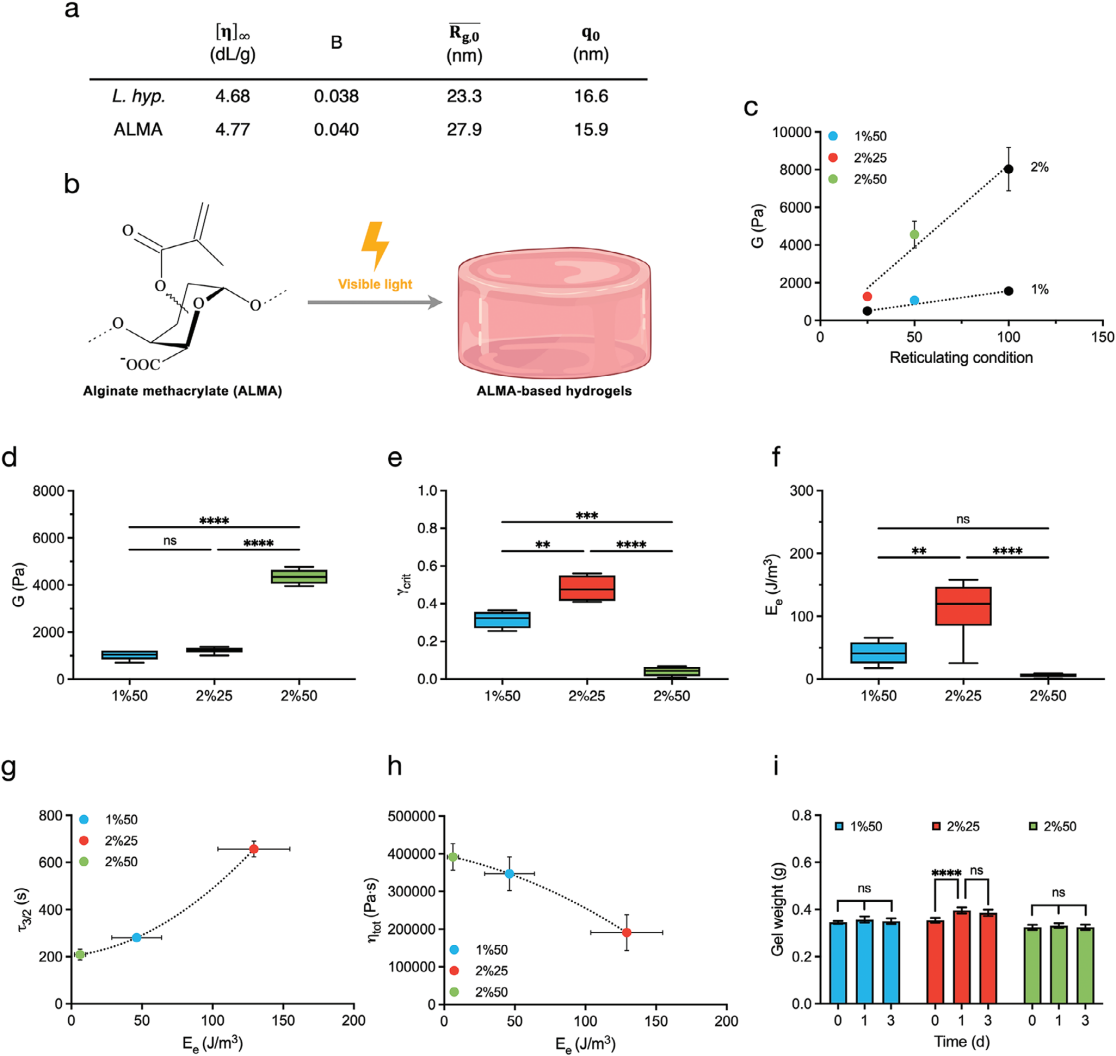
Photopolimerization of ALMA yields hydrogels with tunable mechanical properties. a) Macromolecular parameters determined for alginate from *L. hyperborea* and for ALMA, respectively. [η]_∞_ is the intrinsic viscosity at infinite ionic strength calculated using Equation S11 (Supporting Information). B represents the Smidsrød's rigidity parameter as determined by means of Equation S12 (Supporting Information). $\bar{R_{g,0}}$ and *q*_0_ refer to the radius of gyration (Equation S13, Supporting Information) and the persistence length (Equation S14, Supporting Information), respectively, calculated at infinite ionic strength. For equations, see Appendix SA2, Supporting Information. b) Structure of methacrylated alginate (ALMA) used for the preparation of hydrogels formed by photoreticulation with visible light. Created in BioRender.com. c) Dependence of the shear modulus from reticulating condition (Table S2, Supporting Information) for photocrosslinked hydrogels from ALMA at a final polymer concentration of 1% (w/V) and of 2% (w/V), respectively. See Tables S2 and S3 (Supporting Information), for additional details. d) Shear modulus, G, for the photocrosslinked hydrogels from ALMA. Data obtained from averaging (*n* = 8) the shear modulus from mechanical spectra (Appendix SA3, Supporting Information) and from long stress sweep (Appendix SA4, Supporting Information) (*ns* = not significant, ^****^p < 0.0001 by one‐way ANOVA followed by Tukey's multiple comparison test). e) Critical strain and f) elastic energy (*E_e_*) for the photocrosslinked hydrogels from ALMA. Data obtained from averaging (*n* = 4–6) the data from long stress sweep (Appendix SA4, Supporting Information) (*ns* = not significant, ^**^p < 0.01, ^***^p < 0.001, ^****^p < 0.0001 by one‐way ANOVA followed by Tukey's multiple comparison test). g) Dependence of creep time, τ_3/2_ and h) total viscosity determined from the creep compliance experiments from elastic energy, *E_e_* (Appendix SA5, Supporting Information). Data are reported as mean ± s.d. (*n* = 3). i) Swelling of the hydrogels (Table S3, Supporting Information) in complete DMEM at different time points (*n* = 3, *ns* = not significant, ^****^p < 0.0001 by two‐way ANOVA followed by Tukey's multiple comparison test).

Based on these preliminary experiments, we selected a methacrylated alginate, hereafter referred to as ALMA, containing 6.7% methacrylate groups for further experiments. The reaction conditions did not cleave the polysaccharide backbone of ALMA, which had a molar mass of 190.7 kDa (±1.8 kDa) as determined by SEC‐MALS, nor did they alter its persistence length compared to the native alginate from *L. hyperborea*. The two polysaccharides also exhibited the same [η] versus $I^{-1/2}$ relationship (Appendix SA2, Supporting Information) (**Figure** **1**a).^[^ ^25^, ^26^ ^]^ Therefore, ALMA seems to be a suitable candidate to provide a platform for exploring its applications in 3D cell culture systems.

### 2.2 ALMA Hydrogels are Obtained with Tunable Shear Modulus, Critical Strain, and Elastic Energy

Photocrosslinked hydrogels of ALMA were prepared in an aqueous solution by irradiation with visible light using the photosensitizer eosin Y, the initiator triethanolamine (TEOA), and the catalyst 1‐vinyl‐2‐pyrrolidinone (NVP) (Figure 1b and Table S2 (Supporting Information), for the concentration of reticulating agents used). The shear modulus (Appendix SA3, Supporting Information) of the ALMA hydrogels depended significantly on both the alginate concentration and the amount of reticulating agents. Increasing the concentration of reticulating agents increased the shear modulus. However, the latter effect was more pronounced when the concentration of ALMA in the solution was increased (Figure 1c).

After the initial screening, we selected three conditions, namely 1%50, 2%25, and 2%50 (Table S3, Supporting Information), and characterized the hydrogels further. The shear modulus, G, was determined both by fitting the mechanical spectra with a generalized Maxwell model (Appendix SA3, Supporting Information) and by fitting the long stress sweep data (Appendix SA4 and Figure S3, Supporting Information), obtaining consistent results that were averaged (Figure 1d). Two of the hydrogels from Table S3 (Supporting Information) showed approximately the same shear modulus (≈1 kPa), while the hydrogel prepared in the presence of 2% ALMA and condition 50 of reticulating agents was stiffer (G ≈ 4 kPa).

The three hydrogels from Table S3 (Supporting Information) differed also in terms of the critical strain, γ_*crit*_, which marks the onset of the non‐linear behavior (Figure 1e and Appendix SA4, Supporting Information). In particular, the use of a higher amount of reticulating agents, as in condition 50 in Table S2 (Supporting Information), resulted in the hydrogel being less elastic and deviating from the linear stress‐strain relationship at lower values of strain. This became more apparent when a 2% (w/V) concentration of ALMA was used (sample 2%50 in Table S3, Supporting Information). Conversely, using condition 25 of reticulating agents resulted in a more elastic hydrogel.

The three hydrogels considered (Table S3, Supporting Information) also differed in elastic energy, *E_e_*, which is defined as the total energy stored by the material in the linear deformation range (Figure 1f). The latter ranges from γ = 0 to γ_*crit*_ (Equation 1).^[^ ^22^ ^]^ If the energy transferred to the material exceeds *E_e_*, the material is permanently deformed.^[^ ^22^ ^]^

(1) $$E_{e}=\int_{\gamma =0}^{\gamma_{crit}}\left({\left.\frac{d\sigma }{d\gamma }\right|}_{\gamma =0}\right)\;\gamma d\gamma =\frac{1}{2}\;\;G{\gamma_{crit}}^{2}$$

where σ stands for the stress, γ for the strain and *G* for the shear modulus. The hydrogel with condition 25 in reticulating agents had the highest elastic energy and is therefore more elastic and less prone to permanent deformation. Conversely, both hydrogels with condition 50 in reticulating agents required less energy to be permanently deformed.

Creep compliance analyses showed plastic deformation of the photoreticulated hydrogels from Table S3 (Supporting Information) when the load was maintained over time (Appendix SA5, Supporting Information). A comparison between the three hydrogel samples can be made using the creep time, i.e., τ_3/2_, which is defined as the time for the strain to reach 150% of its original value under a constant stress^[^ ^18^ ^]^ (Appendix SA5, Supporting Information). τ_3/2_ showed a good correlation with *E_e_* (Figure 1g): hydrogels with the reticulation condition 50 showed a rather low τ_3/2_, while the same increased when the reticulating agents were reduced to 25 (Figure S21, Appendix SA5, Supporting Information). In agreement with these considerations, the viscous contribution of the hydrogel with the higher *E_e_* was lower than the other ones, again indicating a higher elasticity of the former compared to the latter (Figure 1h).

We tested the swelling of the three hydrogels from Table S3 (Supporting Information) in complete DMEM for 3 days (Figure 1i). When using the reticulation condition 50, no swelling occurred over time. For the hydrogel with reticulation condition 25, i.e., 2%25, only limited swelling was measured, with a mass increase of ≈9% after 1 day. The weight of the hydrogel remained then unchanged until day 3. We concluded that all hydrogels were stable and showed negligible swelling. Overall, these results emphasize the ability to tailor the mechanical properties of ALMA hydrogels for specific applications in 3D cell culture systems.

### 2.3 Hydrogel Elastic Energy, *E_e_*, Triggers Cellular Stress

**Figure 2:**
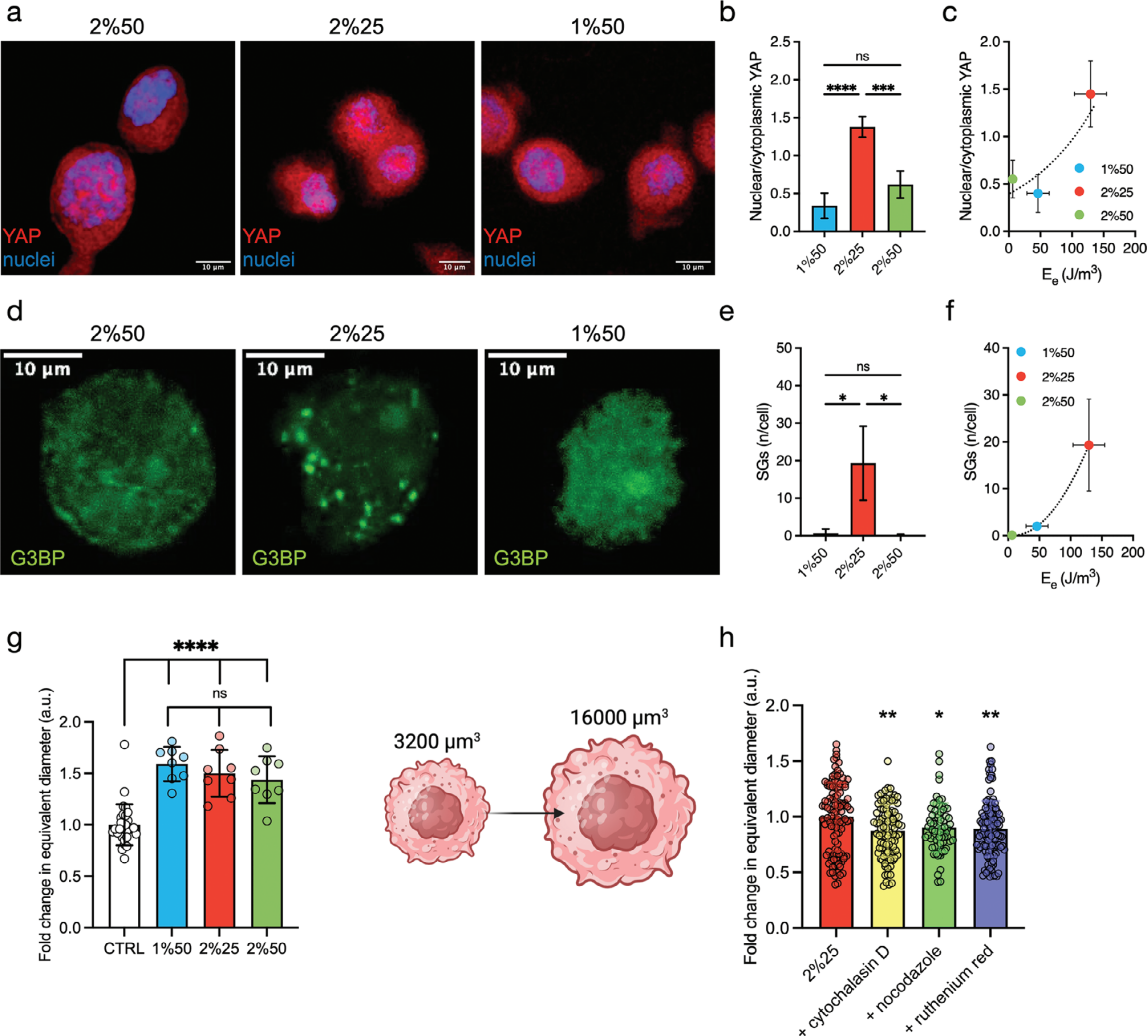
The elastic energy of the hydrogel promotes a cellular stress state and influences YAP translocation, which is mediated by cation channels and cytoskeletal tension. a) Immunostaining of YAP and nuclei and b) ratio between nuclear and cytoplasmic YAP localization (*n* = 4, *ns* = not significant, ^***^p < 0.001, ^****^p < 0.0001 by one‐way ANOVA followed by Tukey's multiple comparison test). Scale bar: 10 µm. c) Dependence of YAP localization from elastic energy, *E_e_*. d) Immunostaining of stress granules using anti‐G3BP antibody and e) ratio between number of stress granules (SGs) per cell (*n* = 3, *ns* = not significant, ^*^p ≤ 0.05 by one‐way ANOVA followed by Tukey's multiple comparison test). Scale bar: 10 µm. f) Dependence of the number of SGs per cell from the elastic energy, *E_e_*. g) Fold change in the equivalent diameter of MG‐63 encapsulated in the hydrogels of Table S3 (Supporting Information) (*n* = 8, *ns* = not significant, ^****^p ≤ 0.0001 by one‐way ANOVA followed by Tukey's multiple comparison test) and graphical representation of cell volume expansion. Cartoon created in BioRender.com h) Fold change in the equivalent diameter of MG‐63 encapsulated in 2%25 hydrogels and treated with cytochalasin D (20 µm), nocodazole (10 µm) or ruthenium red (30 µm) (*n* = 113–140, ^*^p ≤ 0.05, ^**^p ≤ 0.01 by one‐way ANOVA followed by Dunnett's multiple comparison test). In all cases, MG‐63 on day 1 from encapsulation in hydrogels of Table S3 (Supporting Information), was analyzed (cell density = 3 × 10^6^ cells mL^−1^).

To assess the effect of the elastic energy, *E_e_*, on cells, we encapsulated MG‐63 cells in the three selected hydrogels (Table S3, Supporting Information). First, we examined the subcellular localization of YAP after 24 h from MG‐63 encapsulation. YAP and TAZ, key effectors of the Hippo pathway, are known to act as mechanotransducers, transmitting cytoskeletal tension to the nucleus and regulating cellular functions.^[^ ^27^, ^28^ ^]^ More importantly, it is well demonstrated that YAP and TAZ are essential for the biological outcomes of mechanical signals. In the present case, the results of the quantification of YAP subcellular localization confirmed the data reported in the literature, as the YAP immunofluorescence signal was predominantly measured in the nuclei under all conditions tested (**Figure** **2**a). However, there was a significant difference between the three hydrogels, favoring the 2%25 hydrogel for nuclear localization of YAP (Figure 2b) and showing a correlation between YAP nuclear localization and the elastic energy of the ECM mimic (Figure 2c).

Given the limited understanding of how elastic energy, *E_e_*, impacts cells in 3D microenvironments, we next focused on its role on cell response. Elastic energy, in biological terms, can be viewed as the mechanical feedback that cells experience in response to their expansion. This condition of cellular constriction led us to hypothesize a potential connection to cellular stress. Recent evidence suggested that Ras GTPase‐activating protein SH3 domain‐binding protein 1 (G3BP1)‐mediated formation of stress granules (SGs) is partially regulated by mechanical forces.^[^ ^29^ ^]^ SGs are phase‐separated cytoplasmic ribonucleoprotein assemblies that protect RNA and are present when cellular stress occurs.^[^ ^30^ ^]^ For this reason, we first analyzed the formation of stress granules (SGs) on day 1 from cell encapsulation in the ALMA hydrogels. The results of immunohistochemistry with an anti‐G3BP antibody showed a clear difference between the three conditions. While in the 1%50 and 2%50 hydrogels the anti‐G3BP antibody signal appeared almost evenly distributed in the cytoplasm, the 2%25 sample showed a clear presence of SGs (Figure 2d,e). The formation of SGs and hence cellular stress correlated with elastic energy (Figure 2f) of the hydrogels, rather than with the shear modulus (Figure S4, Supporting Information). To confirm this interesting finding, we compared the 2%25 and 1%50 hydrogel conditions, which exhibit the same shear modulus but different elastic energy, and checked qualitatively the formation of cytoplasmic granules based on acidic components after toluidine blue staining (Figure S5, Supporting information). Interestingly, we found that the granules appeared mainly under the 2%25 conditions. Although the latter result is not specific for the type of G3BP‐stress granules, it indirectly supports the presence of organized cytoplasmic granules. Next, to rule out the possibility that the stress granules were associated with cellular damage, cell metabolism was assessed using the AlamarBlue assay, and mitochondrial damage was evaluated with the CCK‐8 assay (Figure S6, Supporting Information). In both cases, no significant differences were observed between the three hydrogels after 24 h, despite the different levels of stress granule formation.

Taken together, these results indicate that the elastic energy of the microenvironment has an impact on the nuclear translocation of the mechanocontroller YAP and, strikingly, on the definition of a stress state for the cells, without affecting cell metabolism and viability.

Next, we wondered whether cells once encapsulated within the ALMA gelling systems, might probe and respond to the mechanical properties of their surrounding microenvironment. Recent research has shown that, in the absence of integrin‐binding ligands and compliant mechanics of the ECM mimic, cells undergo a volumetric expansion as a strategy to sense and adapt to the mechanical properties of the surrounding microenvironment.^[^ ^18^ ^]^ This intriguing behavior suggests that cells may compensate for the lack of direct integrin‐mediated interactions by physically increasing their surface area to maximize contact with the surrounding matrix. To explore this phenomenon, we conducted an initial investigation into the volume expansion of the cells 24 h after encapsulation in the ALMA hydrogels. Our analysis revealed a significant increase in cell diameter compared to matrix‐free cultured MG‐63, with a volumetric expansion approximately four‐fold greater in the hydrogel microenvironment (≈3200 vs 16 000 µm^3^) and, curiously, with negligible differences between the three hydrogel systems investigated (Figure 2g). Since MG‐63 expanded similarly in different ALMA hydrogels, we selected the 2%25 hydrogel condition, namely the more elastic, as model to examine cell volume variation upon treatment with ruthenium red, a well‐known inhibitor of TRPV4 and Piezo channels.^[^ ^31^, ^32^ ^]^ The activation of these mechanosensitive ion channels is crucial for enabling cells to detect and respond to mechanical changes in their environment. Blocking these cation channels resulted in a marked reduction in the volume of encapsulated cells, confirming their crucial role in regulating cell size in response to mechanical stimuli (Figure 2h). Additionally, we explored the involvement of cytoskeleton in this volumetric increase. When small molecule inhibitors of actin (cytochalasin D) and microtubule polymerization (nocodazole) were used, a significant reduction in cell volume was observed (Figure 2h). Collectively, these findings prompted us to speculate that cells within our hydrogels perceive the elastic properties of their surrounding microenvironment through a mechanosensing mechanism that involves cell volume expansion, and this process may be driven by the coordinated action of cation channels and the cytoskeleton tension.

### 2.4 *E_e_* Stressor Triggers ALP Activity

**Figure 3:**
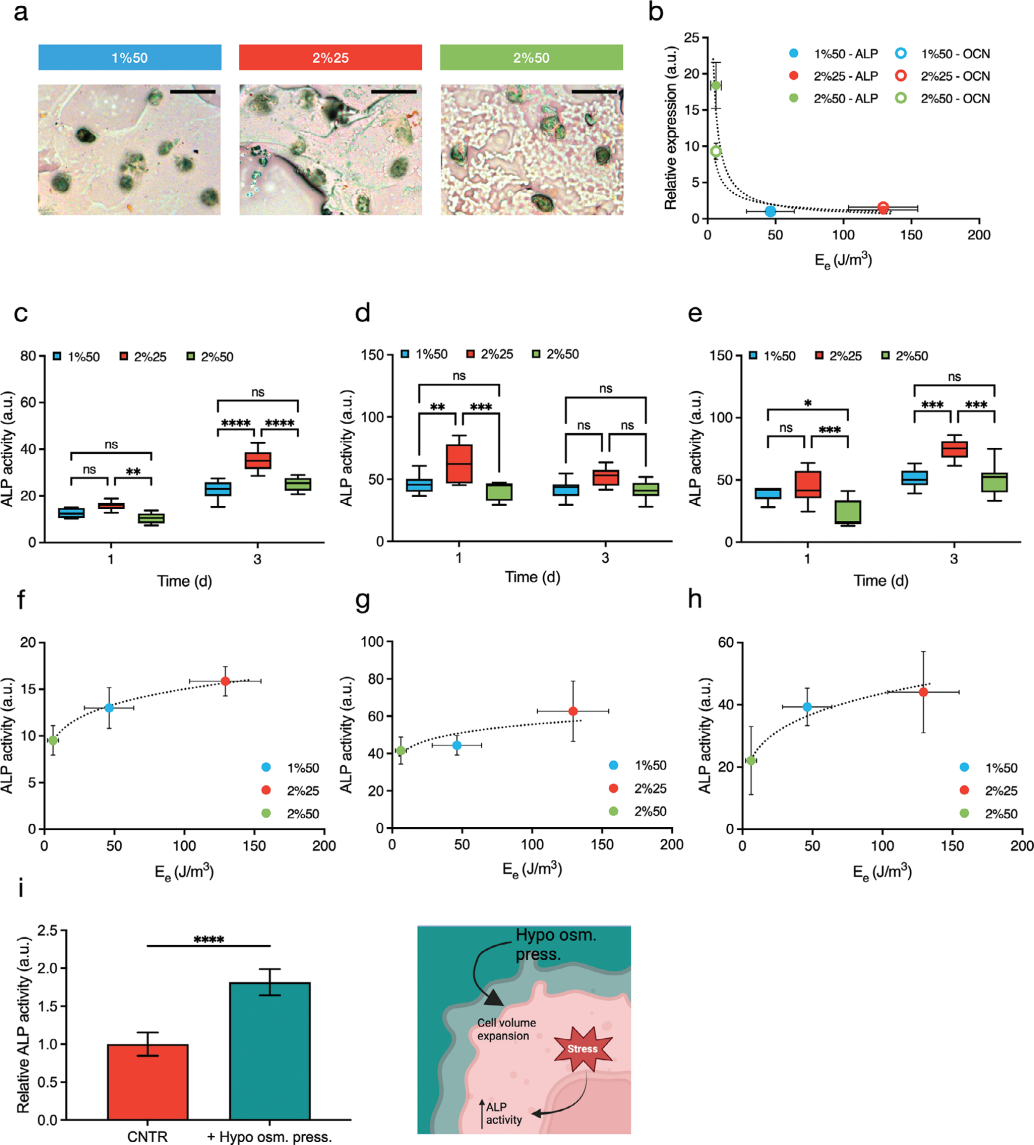
Cellular stress promotes ALP activity. a) Image of MG‐63 cells positive to ALP, detected by means of Fast Blue staining, on day 1 from encapsulation in the three hydrogels of Table S3 (Supporting Information) (cell density 3 × 10^6^ cells mL^−1^). Scale bar: 50 µm. b) Dependence of gene expression of ALP (filled symbols) and of OCN (open symbols) from the elastic energy, E_e_. c–e) ALP activity of MG‐63 encapsulated in hydrogels of Table S3 (Supporting Information) on day 1 and day 3 at a cell density of 1 × 10^6^ cells mL^−1^ (c), 3 × 10^6^ cells mL^−1^ (d), and 7 × 10^6^ cells mL^−1^ (e), respectively (*n* > 7, *ns* = not significant, ^**^p < 0.01, ^***^p < 0.001, ^****^p < 0.0001 by two‐way ANOVA followed by Tukey's multiple comparison test). f–h) Correlation between ALP activity of MG‐63 on day 1 from encapsulation and hydrogel elastic energy, *E_e_*, for a cell density of 1 × 10^6^ cells mL^−1^ (f), 3 × 10^6^ cells mL^−1^ (g), and 7 × 10^6^ cells mL^−1^ (h), respectively. i) Relative ALP activity of MG‐63 in hydrogel 2%25 of Table S3 (Supporting Information) on day 1 from encapsulation (cell density 3 × 10^6^ cells mL^−1^) in hypoosmotic condition. Values are reported with respect to ALP activity of MG‐63 treated with complete DMEM not supplemented with osteogenic differentiation factors at the same time point (*n* = 6, ^****^p < 0.0001 by *t*‐test). Cartoon created in BioRender.com.

There is ample evidence for the involvement of YAP in the osteogenic commitment process of primary osteoblasts and various adult stem cell types, with mechanical stimuli being one of the contributing factors.^[^ ^27^, ^33^ ^]^ Several authors reported that nuclear localization of YAP correlates with the expression and activity of alkaline phosphatase (ALP) and consequently with osteogenic commitment/differentiation.^[^ ^34^, ^35^, ^36^, ^37^ ^]^ In this study, we used human MG‐63 cells from osteosarcoma as a model to demonstrate the influence of cellular stress induced by elastic energy on ALP activity in the absence of differentiation factors. From a qualitative point of view, Fast Blue staining at 24 h confirmed that MG‐63 showed ALP activity when encapsulated in all hydrogels (**Figure** **3**a).

This result prompted us to move forward with the investigation of the early gene expression of two osteoblastic differentiation markers, alkaline phosphatase (ALP) and osteocalcin (OCN),^[^ ^38^, ^39^ ^]^ in MG‐63 cells^[^ ^40^ ^]^ encapsulated in the three ALMA hydrogels on day 1 and in absence of differentiation factors. Our analysis revealed that the expression levels of both ALP and OCN remained substantially low or unchanged in hydrogels with higher elastic energy, particularly in the 1%50 and 2%25 hydrogels (Figure S7, Supporting Information). This observation suggested a potential inverse correlation between gene transcription and *E_e_* for both ALP and OCN. Specifically, the higher the elastic energy, the lower the gene transcription (Figure 3b and Figure S8, Supporting Information).

Considering the impacts of stress granule assembly on gene expression, it is plausible that the observed decrease in ALP and OCN expression could be attributed to the sequestration of mRNA molecules within these dynamic ribonucleoprotein complexes, known to selectively accumulate translationally suppressed mRNAs, leading to decreased mRNA expression levels of genes involved in various cellular processes. Thus, the inverse correlation between gene expression and elastic energy may reflect the assembly of SG in response to cellular stress induced by higher elastic energy conditions. These findings highlight the potential role of these complexes as mediators of gene expression regulation in response to mechanical cues, such as elastic energy.

Next, to investigate the effect of the elastic energy of the ALMA hydrogels on MG‐63 cells, ALP activity was analyzed on day 1 and on day 3 from hydrogel encapsulation, using different cell densities, namely 1 × 10^6^ cells mL^−1^, 3 × 10^6^ cells mL^−1^ and 7 × 10^6^ cells mL^−1^ (Figure 3c–e). In all cases, the 2%25 hydrogel appeared to boost ALP activity at both day 1 and day 3, compared to the other hydrogel formulations. The results on day 1 showed that the ALP activity clearly correlated with the elastic energy, *E_e_*, of the three different materials (Figure 3f–h). While the same trend holds on day 3 from hydrogel encapsulation, the correlation is slightly less stringent (Figure S9, Supporting Information). Nonetheless, a marked decrease in ALP activity was measured after 7 days of incubation, with neither differences among the three hydrogels nor correlation with *E_e_* (Figures S10 and S11, Supporting Information). Interestingly, when MG‐63 were encapsulated and treated with osteogenic factors,^[^ ^41^, ^42^ ^]^ ALP activity was found to be consistent in the three hydrogel systems from Table S3 (Supporting Information) and increased over a period of 7 days (Figure S12, Supporting Information). The low activity of alkaline phosphatase in the presence of osteogenic factors during the early phase of osteogenic commitment may be part of the cellular mechanism that prepares the cells for full osteogenic differentiation, as supported by day 7 results (Figure S12, Supporting Information).^[^ ^8^, ^43^ ^]^ Overall, these results suggest that alkaline phosphatase activity shows a good correlation with elastic energy‐induced cellular stress.

To further strengthen the relationship between the cellular stress state and ALP activity, we chose the 2%25 hydrogels as a matrix model and monitored ALP activity after 24 h using an hypoosmotic medium^[^ ^17^ ^]^ (Figure 3i). By forcing cellular expansion and consequently increasing the mechanical feedback the cell receives from its surrounding environment, ALP activity – which has been shown to correlate with elastic energy – should be enhanced. As shown in Figure 3i, a significant increase in ALP activity was observed, confirming its strong correlation with elastic energy.

## 3 Discussion

In the present work, viscoelastic hydrogels were prepared starting from an alginate modified with methacrylate groups. The degree of substitution was adjusted by varying the amount of reactants, temperature, and reaction time and the reaction was scaled up to 20 grams. A methacrylated alginate, named ALMA, was obtained with a degree of substitution of ≈6.7% and no change in the main macromolecular parameters compared to the unmodified alginate from *L. hyperborea* (Figure 1a). The chemical crosslinking of ALMA induced by visible light makes it possible to obtain elastic hydrogels with a linear stress‐strain range of up to 50% in strain (Figure 1e).

From preliminary experiments, we focused on three soft hydrogels (Table S3, Supporting Information) that have a shear modulus of less than 5 kPa. Samples 1%50 and 2%25 show a very similar shear modulus ≈1 kPa, while sample 2%50 is stiffer with a G of ≈4 kPa (Figure 1d). These values are consistent with those of alginate‐based hydrogels regulating cartilage matrix formation.^[^ ^18^ ^]^ The average mesh size did not show significant differences between the three hydrogels of Table S3 (Supporting Information) and ranged from 20 to 12 nm (Appendix SA6, Supporting Information).

Apart from the shear modulus, the three hydrogels differed in the extent of their elastic response prior to permanent deformation. In particular, a reticulating condition 50 (Table S2, Supporting Information) resulted in the formation of brittle hydrogels, with a low value of critical strain and elastic energy, indicating that little energy is required for plastic modification. In contrast, the 2%25 hydrogel with a lower amount of reticulating agents was more elastic, with a higher elastic energy and critical strain. This conclusion is also supported by the analysis of τ_3/2_, i.e., the time required for the strain to reach 150% of the original value in a creep compliance measurement, or by the viscosity of the hydrogels. Samples with reticulating conditions 50 show a τ_3/2_ in the order of 300 s, while a longer creep time (≈700 s) characterized the hydrogel 2%25 (Figure 1g). In general, all hydrogels considered were by far more elastic than calcium alginate hydrogels, which have a τ_3/2_ of ≈100 s.^[^ ^26^ ^]^ In the present case, there is a correlation between the creep time and elastic energy. However, this is not always the case and *E_e_* can be depicted as a more general parameter describing the extent of elastic deformation for the materials.^[^ ^12^ ^]^

Next, we focused on the effect of the hydrogel on the encapsulated MG‐63. Mechanical confinement influences cell responses in different ways. As an example, fast‐relaxing hydrogels increase the formation of cartilage matrix by chondrocytes while cartilage degradation and cell death are associated with slower‐relaxing hydrogels.^[^ ^18^ ^]^ In a 3D environment, cells have two pathways of mechanosensing: i) the actin‐myosin‐integrin axis in which binding motifs such as the RGD peptide play a role and promote cell spreading, and ii) the osmotic swelling, in which the cells increase their volume – still retaining a rounded shape – by an influx of medium. Both pathways can be active when a network decorated with adhesive peptides is used,^[^ ^44^ ^]^ and distinguishing the consequences of each of these pathways can be puzzling. In the present manuscript, we focused on the second pathway. To this end, the three hydrogels used were not chemically coupled with the RGD motif, and the mechanosensing by the cells is not mediated by integrins, since alginate is non‐adhesive.^[^ ^45^ ^]^ It has been reported that cells sense viscoelastic properties of the microenvironment through variations in cell volume^[^ ^18^, ^20^ ^]^ and that its regulation influences various cellular processes, such as stem cell differentiation.^[^ ^20^ ^]^ A small dependence of cell volume on relaxation time has been reported, especially for fast‐relaxing hydrogels,^[^ ^18^ ^]^ while a correlation^[^ ^46^ ^]^ between cell volume and hydrogel stiffness was previously found. Suspended, non‐encapsulated MG‐63 exhibited a mean cell diameter of ≈19.8 µm, with a cell volume of ≈3200 µm^3^. Regardless of the ALMA hydrogel used, the encapsulated MG‐63 exhibited a four‐fold increase in volume (Figure 2g), resulting in a substantial strain on the hydrogel that likely causes its plastic deformation. The dimensions of MG‐63 are not significantly different from those of mesenchymal stem cells encapsulated in a 3D environment, demonstrating that volume expansion is likely a common way in which the cells perceive their environment.^[^ ^44^ ^]^

It is intriguing that YAP localization between nucleus and cytoplasm parallels the elastic energy of the three hydrogels considered. The transcriptional regulators YAP and TAZ play a fundamental role in how cells perceive their physical microenvironment.^[^ ^27^ ^]^ So far, however, the translocation of YAP/TAZ has been correlated with the stiffness of the matrices in which the cells are embedded. It has been reported that the 2D cell cultures on viscoelastic materials undergo less elongation at a given load, resulting in lower stress and a lower YAP/TAZ N/C ratio.^[^ ^47^ ^]^ In 3D matrices, the localization of YAP/TAZ shows an opposite trend, as already reported in the literature.^[^ ^44^ ^]^ Lee et al. found that fast‐relaxing hydrogels reduce cell confinement and increase YAP activation.^[^ ^17^ ^]^ Major et al. found a correlation between the volume of the cells, the stiffness of the matrix, and the localization of YAP.^[^ ^46^ ^]^ However, in the latter two cases, the cells were embedded in a 3D hydrogel system containing cell adhesion motifs and spreading occurred. An effect of elastic energy on translocation of YAP on adhering cells in a 2D environment has recently been reported, with more dissipative (more elastic) matrices leading to a lower localization of YAP in the nucleus than in the cytoplasm.^[^ ^12^, ^48^ ^]^ In the present work, the hydrogel with higher elastic energy shows the greater localization of YAP (Figure 2a–c) in the nucleus of cells and no correlation was found with their volume. This strengthens the role of *E_e_* as a mechanical trigger for the cells.

The hydrogels embedding MG‐63 oppose to cell volume expansion causing cellular stress: the lower the compliance of the matrix, the higher the cellular stress condition detected as presence of SGs. The latter correlated with elastic energy rather than shear modulus, pointing to elasticity, described as resistance to permanent (plastic) deformation, as fundamental element for cellular stress. Plastic deformation has been previously reported as a key factor in determining cell adhesion in 2D, with a threshold above which no adhesion is noticed even in the presence of a fibronectin coating.^[^ ^22^ ^]^ While the shear modulus represents the resistance of the hydrogel in the small deformation range, to permanently deform the matrix cells need to extend the deformation to the non‐linear stress‐strain range, where shear modulus decreases (strain softening).^[^ ^22^ ^]^ A strong correlation was seen in the present system for 3D encapsulation: when the stressor *E_e_* is high, i.e., for 2%25, cells experience a stress condition with the formation of SGs (Figure 2d–f).

The pathway for SGs formation is intertwined with the Unfolded Protein Response (UPR): SGs formation is a consequence of UPR induction.^[^ ^49^ ^]^ UPR is a feedback loop that aims to mitigate cellular stress and reestablish homeostasis or induce apoptosis.^[^ ^50^ ^]^ This confirms the pivotal role of UPR in various human diseases controlling cell death when homeostasis fails.^[^ ^50^ ^]^ When operating in the endoplasmic reticulum, UPR is an adaptive response counteracting the accumulation of unfolded proteins.^[^ ^51^ ^]^ Activation of UPR leads to a modification of gene expression, rate of protein folding, maturation, and trafficking.^[^ ^52^ ^]^ This is found in the strong correlation between SGs, ALP, and OCN gene expression, and ALP activity with the elastic energy (*E_e_*) of the hydrogels (Figures 2f, 3b and 3f–h) in the first day of incubation. Elastic energy is than a stressor that activates cellular stress, likely inducing an Unfolded Protein Response (UPR), with two‐fold consequences: decrease in gene expression and increase in ALP proper folding.^[^ ^52^ ^]^ Additional experiments are needed to confirm or exclude this.

From day 3, cells start to adapt with a mitigation of the stress detected as a partial loss in correlation between ALP activity and *E_e_* (Figure S9, Supporting Information). At day 7, the ALP activity is markedly reduced, and no differences are detected among the three hydrogels (Figures S10 and S11, Supporting Information). Focusing on 2%25 hydrogel, an additional increase in cellular stress, obtained through the use of an hypoosmotic medium, led to an increase in ALP activity (Figure 3i), confirming the proposed link between the two phenomena.

A correlation between ALP gene expression and the shear modulus of the hydrogels was recently reported in 2D.^[^ ^8^ ^]^ Similarly, OCN gene expression and ALP activity correlate in BM‐MSCs treated with differentiation agents in 3D‐printed scaffolds.^[^ ^53^ ^]^ ALP gene expression was correlated with the modulus of human adipose tissue‐derived stem cells incubated for 21 days on PCL‐coated scaffolds in osteogenic medium.^[^ ^54^ ^]^ In contrast, ALP activity did not correlate with the compressive modulus for MC3T3 encapsulated and treated in the presence of differentiation agents, at least in the first days.^[^ ^55^ ^]^ A discrepancy between gene expression and calcium deposition was recently found by Žigon‐Branc et al.^[^ ^56^ ^]^ using human adipose tissue‐derived stem cells encapsulated in methacrylated gelatin hydrogels. While ALP gene expression correlated with the storage modulus of the hydrogel, with lower expression observed in the softer hydrogels, calcium deposition showed an opposite correlation and was more pronounced in the softer hydrogels. The behavior we found for encapsulated MG‐63 in ALMA‐based hydrogel closely parallels that of Žigon‐Branc et al.^[^ ^56^ ^]^ Defining *E_e_* as a stressor inducing cellular stress reconciles the apparent discrepancies and provides a novel insight in mechanosensing by cells in 3D environment.

**Figure 4:**
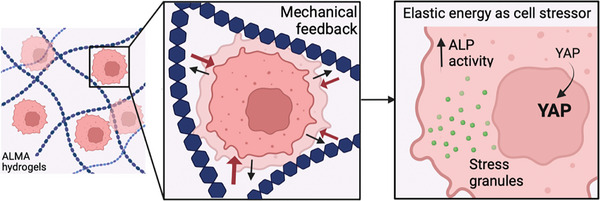
Schematic cartoon summarizing what was identified in this study. ALMA‐based hydrogels with tunable linear stress‐strain response under oscillatory shear, i.e., elastic energy, were used as a 3D cell culture model to study the effects of elastic energy on cell behavior. As the elastic energy of the matrix increases, along with the associated higher mechanical feedback, cellular stress also increases. This correlates with the formation of G3BP‐mediated stress granules, which is accompanied by an increase in alkaline phosphatase (ALP) activity and nuclear translocation of Yes‐associated protein (YAP), but a decrease in gene expression. Created in BioRender.com.

In conclusion, the present study has shed light on the role of elastic energy in mediating cellular responses in 3D microenvironments (**Figure** **4**). Of note, elastic energy emerges as a critical mechanical property that cells sense during their expansion. As cells push against the hydrogel matrix, they experience mechanical feedback in the form of elastic energy. Interestingly, this mechanical feedback seems to induce a state of cellular stress, possibly due to the strain experienced by the cells as they adapt to the constraints of the hydrogel environment. However, it is important to acknowledge certain limitations that should be considered when interpreting the results. The study primarily focuses on a specific cell line and its response to elastic energy, and caution should be taken when extrapolating the results to other cell types. Moreover, while correlations between elastic energy, cellular stress, and specific cellular processes have been established, the underlying molecular mechanisms remain elusive. Further investigations are needed to unravel the intricate signaling pathways involved. Despite these limitations, our study provides valuable insights into the role of elastic energy as a stressor and lays the groundwork for future research in this field. The novel hydrogel design, detailed mechanical characterization, and investigation of cell response contribute to the growing body of knowledge in mechanobiology, tissue engineering, and regenerative medicine, with implications for the development of biomaterials and therapeutic strategies.

## 4 Experimental Section

### Materials

Alginate from *L. hyperborea* (F_G_ = 0.69; F_GG_ = 0.56; average molecular weight (Mw) = 114 kDa; PI = 1.9) was kindly provided by FMC BioPolymer AS (Sandvika, Norway). Methacrylic anhydride, 1‐vinyl‐2‐pyrrolidinone, eosin Y, mixture of ethanol/isopropanol (ETA/IPA), phosphate‐buffered saline (PBS), sodium hydroxide, triethanolamine, Triton X‐100, AlamarBlue reagent were all purchased from Merck/Sigma‐Aldrich (St. Louis, MO, USA). DMEM (Dulbecco's Modified Eagle's Medium) High Glucose, Fetal Bovine Serum (FBS), streptomycin, penicillin, and trypsin were from EuroClone (Italy).

### Synthesis of Methacrylate Alginates

Methacrylic anhydride was added to an aqueous solution of sodium alginate in the presence of NaOH at different temperature. The slurry was stirred for different times and added of an aqueous NaOH solution (5 m) to reach a basic pH value. The product was precipitated by dropwise addition of a mixture of ethanol/isopropanol (ETA/IPA). The product was extensively washed with increasing mixtures of water and ETA/IPA from 50% to 100%. The product was filtered and dried at 60 °C under reduced pressure. The product was analyzed by means of ^1^H‐NMR. The amount of reactants used in the different synthesis is reported in Table S1 (Supporting Information).

### Synthesis of ALMA

Sodium alginate (15 g) was dissolved in deionized water (375 mL). A solution of NaOH (15 g) dissolved in deionized water (75 mL) was added followed by the dropwise addition of methacrylic anhydride (120 mL). The slurry was vigorously stirred for 60 min and then added of aqueous NaOH 5 M (150 mL). The product was extensively washed with increasing mixtures of water and ETA/IPA from 50% to 100%. The product was filtered and dried at 60 °C under reduced pressure. Yield: 13 g. The degree of substitution was determined as 6.7% as determined by ^1^H‐NMR.

### Nuclear Magnetic Resonance (NMR)

The lyophilized methacrylated OligoG (≈5 mg) was dissolved in 600 µL 99.9% D_2_O (d‐99.9%; Sigma‐Aldrich, Norway) and 2 µL 1% TSP (3‐(Trimethylsilyl)‐propionic‐acid sodium salt; Aldrich, Milwaukee, WI) in D_2_O was added for internal chemical shift reference. All spectra were recorded at 25 °C on a BRUKER AVIIIHD 800 MHz equipped with 5 mm cryogenic CP‐TCI Probe. The spectra were recorded using TopSpin 3.5 pL7 software (Bruker BioSpin) and processed and analyzed with TopSpin 4.0.7 software (Bruker BioSpin).

For chemical shift assignment, the following one‐ and 2D NMR experiments were performed: 1D proton with water suppression, 2D ^1^H‐^1^H TOCSY (total correlation spectroscopy) using a 70 ms mixing time, ^1^H‐^1^H NOESY (nuclear Overhauser enhanced spectroscopy) using an 80 ms mixing time, DOSY (diffusion‐ordered spectroscopy) using a Bruker BioSpin stimulated echo pulse sequence with bipolar gradients where gradient pulses of 2 ms duration (δ) and 32 different strengths varying linearly from 0.03 to 0.57 T·m^−1^ were applied, and the diffusion delay (Δ) was set to 80 ms, ^1^H‐^13^C HSQC (heteronuclear single quantum coherence) with multiplicity editing, ^1^H‐^13^C H2BC (heteronuclear two bond coherence), and ^1^H‐^13^C HMBC (heteronuclear multiple bond coherence) with suppression of one‐bond correlations.

### Intrinsic Viscosity Measurements

Intrinsic viscosity was measured at 20 °C by means of a Schott–Ger¨ate AVS/G automatic measuring apparatus and an Ubbelohde‐type capillary viscometer. An aqueous solution containing different amounts of NaCl was used as solvent. The intrinsic viscosity [η] values were determined by analyzing the polymer concentration dependence of the reduced specific viscosity (η_sp_/c) and of the reduced logarithm of the relative viscosity (ln(η_rel_)/c) using the Huggins (Equation 2) and Kraemer (Equation 3) equations, respectively:

(2) $$\frac{\eta_{sp}}{c}=\left[\eta \right]+k^{\prime }{\left[\eta \right]}^{2}c$$

(3) $$\frac{ln\left(\eta_{rel}\right)}{c}=\left[\eta \right]-k^{\prime \prime }{\left[\eta \right]}^{2}c$$

where k′ and k″ were the Huggins and Kraemer constants, respectively. For each polymer concentration, five replicates were averaged.

### Size Exclusion Chromatography—Multi‐Angle Light Scattering (SEC‐MALS)

ALMA and unmodified alginate were analyzed by Size Exclusion Chromatography (SEC) with Multiangle Light Scattering (MALS). Samples were dissolved in the mobile phase (0.15 m NaNO3, pH 6.0 with 10 mm EDTA) and filtered (0.45 µm) before injection. An Agilent Technologies 1260 Infinity ii IsoPump with a 1260 HiP degasser was used to maintain a flow of 0.5 mL min^−1^ 1 during analyses. The samples (50–100 µL) were injected from an Agilent 1260 Infinity ii vialsampler. Separation was performed on serially connected OHpak LB‐806 and OHpak LB‐805 columns (Shodex). The column outlet was connected to a Dawn Heleos‐II detector (Wyatt) followed by a RI‐501 refractive index detector (Shodex). Astra 7.3.2 software was used for data acquisition and processing. For each sample, three replicates were averaged.

### Composition of ALMA Solutions and Hydrogels Generation

Alginate methacrylate (ALMA) solutions were prepared in PBS 1× buffer, pH 7.4. Briefly, alginate methacrylate (100–200 mg) was solubilized in 8.65 or 8.825 mL of deionized water. Finally, 1 mL of PBS 10× was added to have a final volume of 9.65 or 9.825 mL. The crosslinking solution composed of eosin Y (0.07 m), 1‐vinyl‐2‐pyrrolidinone (9.35 m), and triethanolamine (0.82 m in PBS 1×, pH 8) was mixed with ALMA solutions at various volume ratios at 37 °C overnight, thus resulting in three different ALMA solutions. In all tests, the final experimental conditions were: [ALMA] = 1–2% w/V, [Eosin Y] = 2.8 × 10^−5^–5.7 × 10^−5^ m, [1‐vinyl‐2‐pyrrolidinone] = 0.03–0.067 m, [triethanolamine] = 0.01–0.02 m, respectively. Hydrogels from the different ALMA solutions were obtained by irradiation with visible light (λ = 400–500 nm, 3 m ESPE Curing Light 2500, 230 V, 50/60 Hz) for 1 min at room temperature to allow for photo‐crosslinking. The composition of the three gels is reported in Table S3 (Supporting Information).

### Mechanical Characterization of the Hydrogels

Rheological characterization of hydrogel disks (Ø = 20 mm) was performed by means of a controlled stress rheometer HAAKE MARS III operating at *T* = 37 °C using a shagreened plate‐plate apparatus (“HPP20 *profiliert*”: diameter = 20 mm) as the measuring device. To avoid water evaporation from hydrogels, measurements were performed in a water‐saturated environment formed by using a glass bell (solvent trap) containing a wet cloth. In addition, to prevent both wall‐slippage and excessive hydrogel squeezing, the gap between plates was adjusted by executing a series of short stress sweep tests (*ν* = 1 Hz; stress range 1–5 Pa) until a constant *G*′ was reached. The linear viscoelastic range was determined by means of stress sweep tests consisting in measuring the elastic (*G*′) and viscous (*G*″) moduli variation with increasing shear stress (1 Pa *< τ <*1000 Pa) at a frequency *ν* = 1 Hz (hence with *ω* = 2*𝜋𝜈* = 6.28 rad s^−1^). The mechanical spectra (frequency sweep tests) were recorded by measuring the dependence of the elastic (*G*′) and viscous (*G*″) moduli on pulsation frequency at constant shear stress *τ* = 5 Pa (well within the linear viscoelastic range).

Creep compliance measurements were performed by applying on each hydrogel a constant stress, corresponding to an initial deformation of ≈5%. The increase in strain was monitored for 900 s, the stress was unloaded and the recovery of the strain followed for additional 900 s.

### Cell Culture

Human osteosarcoma MG‐63 (ATCC CRL‐1427) was cultured in Dulbecco's Modified Eagle's Medium High Glucose (EuroClone, Italy), supplemented with 10% heat‐inactivated fetal bovine serum (EuroClone, Italy) and 1% penicillin/streptomycin (EuroClone, Italy) (i.e., complete DMEM), in a humidified atmosphere of 5% CO_2_ at 37 °C.

### Preparation of Cell‐Laden Hydrogels

For the biological in vitro tests, the ALMA solutions were prepared as described in Table S3 (Supporting Information). A precise number of cells were added to reach a final cell density of 1 × 10^6^, 3 × 10^6^ or 7 × 10^6^ MG‐63 cells mL^−1^ of ALMA solution. Finally, 200 µL of the cell‐loaded ALMA solutions were poured into a 96‐well plate and then irradiated with visible light (λ = 400–500 nm 3 m ESPE Curing Light 2500, 230 V, 50/60 Hz) for 1 min at room temperature to generate the cell‐laden hydrogels. After reticulation, the hydrogels were transferred into a 24‐well plate and 1 mL of complete DMEM was added. For osteogenic differentiation assays, hydrogels were cultured in osteogenic induction medium consisting of complete DMEM supplemented with 50 µg mL^−1^ L‐ascorbic acid (Fluka Biochemika), 10 mm β‐glycerophosphate (Fluka Biochemika) and 0.1 µm dexamethasone (Merck/Sigma‐Aldrich, St. Louis, MO, USA).^[^ ^57^ ^]^ For the assay in the absence of osteogenic differentiation factors, hydrogels were cultured in complete DMEM. The medium was changed every 2–3 days.

### Viability Assay

The metabolic activity of MG‐63 cells encapsulated in the hydrogels was assessed using the AlamarBlue assay according to manufacturer's protocol (Merck/Sigma‐Aldrich, St. Louis, MO, USA). The three hydrogels (Table S3, Supporting Information) were prepared as previously described using a cell density of 3 × 10^6^ MG‐63 cells mL^−1^ of ALMA solution. Briefly, at selected time points, the hydrogels were washed with PBS 1× and incubated with 500 µL per well of AlamarBlue reagent diluted 1:30 v/v in fresh complete DMEM. After 4 h of incubation at T = 37 °C, 120 µL of supernatants were transferred into a black 96‐well microtiter plate. The fluorescence intensity was measured using a FLUOStar Omega by BMG‐Labtech spectrofluorometer, with excitation wavelength (λ_ex_) of 544 nm and emission wavelength (λ_em_) of 590 nm. The mixture AlamarBlue‐DMEM was used as blank.

To evaluate any mitochondrial damage, CCK‐8 assay was performed according to manufacturer's protocol (Merck/Sigma‐Aldrich, St. Louis, MO, USA). The three hydrogels (Table S3, Supporting Information) were prepared as previously described using a cell density of 3 × 10^6^ MG‐63 cells mL^−1^ of ALMA solution. Briefly, after 24 h of culture in complete DMEM, the hydrogels were washed with PBS 1× and incubated with 500 µL per well of CCK‐8 reagent diluted 1:10 v/v in fresh complete DMEM. After 2 h of incubation at T = 37 °C, absorbance was measured at 450 nm using TECAN spectrofluorometer. The mixture CCK‐8/DMEM was used as blank.

### Semi‐thin Section of the Hydrogels

The 2%25 and 1%50 cell‐laden hydrogels were prepared as previously described using a cell density of 3 × 10^6^ MG‐63 cells mL^−1^ of ALMA solution. They were then incubated for 24 h in complete DMEM. Then, the hydrogels were fixed in 0.5 mL of 1% paraformaldehyde, 2.5% glutaraldehyde in 0.1 mol L^− 1^ phosphate buffer saline, pH 7.4. The hydrogels were then post‐fixed in 1% osmium tetroxide in the same phosphate buffer saline, dehydrated in ethanol (50%, 70%, 95%, and absolute), and embedded in LR White (Sigma‐Aldrich). A Pabisch TOP Ultra 150 was used to cut semi‐thin resin sections (1 µm) which were stained with toluidine blue and examined with an Olympus BX50; images were acquired with an Panasonic DC‐G100 camera (Olympus Corporation, Shinjuku, Tokyo, Japan) at 100× magnification.

### Cell Morphology Experiment

For cell morphology experiments, MG‐63 cells were initially detached and resuspended in cell culture medium at a density of 1 × 10^6^ cells mL^−1^. Then, 5 µL mL^−1^ of medium of BioTracker 490 Green Cytoplasmic Membrane Dye (Merck/Sigma‐Aldrich, St. Louis, MO, USA) was added in the cell suspension. After 20 min of incubation at 37 °C, cell suspension was centrifuged at 1500 rpm for 5 min and washed thrice with PBS 1× to remove the excess of cell labeling solution. Then, the different cell‐laden hydrogels (Table S3, Supporting Information) were prepared as previously described using a density of 3 × 10^6^ MG‐63 cells mL^−1^ of ALMA solution. Next, 1 mL of each cell‐laden ALMA solution was poured in different chambers of a µ‐Slide 4 chamber (ibidi GmbH, Germany), irradiated by visible light for 1 min and cultured in complete DMEM for 24 h.

For the experiments in presence of chemicals, 2%25 hydrogels were prepared using the same protocol but culturing them in complete DMEM supplemented with cytochalasin D (20 µm, Santa Cruz Biotechnology), nocodazole (10 µm, Merck/Sigma‐Aldrich, St. Louis, MO, USA) or ruthenium red (30 µm, Merck/Sigma‐Aldrich, St. Louis, MO, USA). Images from immunofluorescence and cellular staining experiments were acquired using a Nikon C1si confocal microscope (Nikon, Tokyo, Japan). The light was delivered to the sample with an 80/20 reflector. The system was operated with a pinhole size of one Airy disk. Electronic zoom was kept at minimum values for measurements to reduce potential bleaching. For the different fields collected 20× Plan Fluor objectives were used, saving series of optical images respectively at 707 × 707 µm with 4 µm z‐resolution step size. Images in various conditions were captured under identical acquisition settings to allow comparison of fluorescent intensity and were processed for maximum z‐projection by using Fiji‐ImageJ 2.9.0/1.53t (NIH, Bethesda, USA).

Image analysis to assess the volume and aspect ratio of each identified cell was performed using a custom MATLAB code that implemented the following steps. First, images were rescaled via interpolation to achieve isotropic voxel size, simplifying subsequent numerical assessment. Next, rescaled images were segmented using a manually determined global thresholding approach, incorporating the output of Canny edge detection, which was superimposed onto the thresholded image. Simple morphological filling was then applied to more accurately capture the complete volume of each cell. Connected components (cells) that either had an unrealistic volume (smaller than a custom‐specified value) or were only partially included within the field of view (FOV) were removed from the segmented images. For all remaining objects in each binary image, volume was assessed by counting the number of voxels comprising the connected component (scaled by the voxel size), and the adimensional cell aspect ratio was determined as the ratio between the minimum and maximum widths of the bounding box. MATLAB's regionprops3 command was used for these latter two measurements.

### Alkaline Phosphatase (ALP) Activity Assay

At each time point, hydrogels were collected and washed twice with PBS 1×. Subsequently, 100 µL of cell lysis solution (Tris 100 mm, pH 9.8, and 0.5% Triton X‐100) was added to each sample and then mechanically disrupted to break down the hydrogel. The samples were incubated sequentially for 30 min at *T* = −80 °C and 10 min at *T* = 60 °C. After the samples were transferred to a filtered Eppendorf tube, they were centrifuged at 13 000 × g for 5 min to separate the protein‐containing cell lysate and the hydrogel residues. Enzymatic activity was measured by adding 40 µL of lysate and 40 µL of reaction buffer (Tris 100 mm pH 9.8, 1 mm MgCl_2,_ and 6 mM para‐nitro‐phenyl phosphate) to a 96‐well plate. After 2 h of incubation at 37 °C under dark conditions, the reaction was stopped by adding 2 µL NaOH 5 m, and the absorbance was measured at 420 nm using a FLUOStar Omega from BMG‐Labtech (Germany) spectrofluorometer. The results were expressed by normalizing ALP activity to the amount of protein content quantified by microBCA assay according to the manufacturer's protocol (Merck/Sigma‐Aldrich, St. Louis, MO, USA). For ALP activity assay in hypoosmotic conditions, 2%25 cell‐laden hydrogels were prepared as previously described using a density of 3 × 10^6^ MG‐63 cells mL^−1^ of ALMA solution. The hypoosmotic pressure of the medium was altered by diluting complete DMEM with 20% of deionized water and 1 mL of diluted medium was added to the hydrogels after hydrogels reticulation. After 24 h of incubation in hypoosmotic conditions, ALP activity assay was carried out as previously described.

### Gene Expression Analysis

Quantitative Real‐Time PCR (qPCR) was used to assess the relative expression of OCN and ALP differentiation marker genes. RNA was extracted from cell‐laden hydrogels (cell density = 3 × 10^6^ cells mL^−1^ of ALMA solution) on days 1, 3, and 7 from encapsulation by using TRI‐Reagent (Merck/Sigma‐Aldrich, St. Louis, MO, USA) according to the manufacturer's instructions. For cDNA synthesis, 500 ng of total RNA were retrotranscribed with M‐MLV reverse transcriptase (Thermo Fisher Scientific, Waltham, MA, USA) and hexameric random primers or poly‐(T)20 primer (Merck/Sigma‐Aldrich, St. Louis, MO, USA). Specific primer pairs (Table S4, Supporting Information) were designed to analyze gene expression levels by SYBR Green qPCR (Bio‐Rad Laboratories, Redmond, WA, USA). The expression of the housekeeping gene GAPDH was used to normalize expression of the target genes. qPCR amplifications were performed using a CFX96 Real‐Time PCR detection system (Bio‐Rad Laboratories, Redmond, WA, USA) and the relative expression levels were calculated using the 2^‐∆∆CT method.^[^ ^58^ ^]^

### Cell Immunostaining and Image Analysis

Hydrogels with different composition (Table S3, Supporting Information) were prepared as previously described using a density of 3 × 10^6^ MG‐63 cells mL^−1^ of ALMA solutions. Then, 1 mL of each cell‐laden solution was poured into a 24‐well plate, irradiated by visible light for 1 min. After 24 h incubation in complete DMEM, hydrogels were punched into small disks (6 mm in diameter, 5 mm thick) and moved in different Eppendorf tubes. Hydrogels were fixed with formaldehyde 4% v/v (Merck/Sigma‐Aldrich, St. Louis, MO, USA) in PBS 1× for 20 min at room temperature. Then, hydrogels were washed 5× with PBS 1× and permeabilized with Triton 0.2% v/v (Merck/Sigma‐Aldrich, St. Louis, MO, USA) in PBS 1× for 15 min at room temperature. Next, hydrogels were washed with PBS 1× and incubated with BSA 4% w/v (Merck/Sigma‐Aldrich, St. Louis, MO, USA) + Normal Goat Serum 5% v/v (Merck/Sigma‐Aldrich, St. Louis, MO, USA) in PBS 1× (i.e., blocking mixture) for 1 h at 37 °C. The blocking solution was then removed, and the samples washed with PBS 1×. The following primary antibodies were used for immunostaining: YAP antibody (dilution 1:200, sc‐101199, Santa Cruz), and G3BP antibody (dilution 1:150, 611126, BD Bioscience). Primary antibodies were diluted in blocking mixture. In the case of YAP, Triton 0.1% v/v was also added to blocking mixture. Incubation was proceeded overnight at 4 °C. Then, hydrogels were washed and incubated with secondary antibodies diluted in blocking mixture for 3 h at room temperature. The following secondary antibodies were used for immunostaining: anti‐mouse IgG_k_−CruzFluor™ 647 (dilution 1:250, sc‐516179, Santa Cruz) and anti‐mouse IgG (Fab specific)−FITC antibody (dilution 1:150, F5262, Merck/Sigma‐Aldrich, St. Louis, MO, USA) for YAP and G3BP, respectively. For the visualization of nuclei, cells were counterstained with DAPI (33258, Invitrogen, 5 µg mL^−1^ in PBS 1×). Finally, hydrogels were washed once and stored in PBS 1×. Images from immunofluorescence and cellular staining experiments were acquired using a Nikon C1si confocal microscope (Nikon, Tokyo, Japan). The light was delivered to the sample with an 80/20 reflector. The system was operated with a pinhole size of one Airy disk. Electronic zoom was kept at minimum values for measurements to reduce potential bleaching. For the different fields collected 40× Plan Apo objectives were used, saving series of optical images respectively at 138 × 138 µm with 2 µm z‐resolution step size for YAP immunostaining and 320 × 320 µm with 5 µm z‐resolution step size for G3BP immunostaining. Images in various conditions were captured under identical acquisition settings to allow comparison of fluorescent intensity and were processed for maximum z‐projection by using Fiji‐ImageJ 2.9.0/1.53t (NIH, Bethesda, USA).

### Statistical Analysis and Software

Statistical comparisons were performed with GraphPad Prism 10.2.3 for mac OS, GraphPad Software, Boston, Massachusetts, USA, www.graphpad.com. *T*‐test, one‐ or two‐way ANOVA tests were applied to compare data between groups with a Tukey's or Dunnet's *post hoc* comparison. Differences were considered significant if the *p*‐value was less than 0.05. The cartoons were created in BioRender.com.

## Conflict of Interest

The authors declare no conflict of interest.

## Author Contributions

S.L., P.S., E.M., I.D. conceptualized the study. S.L., P.S., M.C., F.S., M.R., F.B., F.L.A., P.G.G. curated data. I.D., F.L.A. acquired funding. S.L., M.C., F.S., M.R., F.B., P.G.G., F.L.A., I.D. investigated the project. P.S., M.R., F.B., P.G.G., F.L.A., E.M., I.D. developed the methodology. P.S., E.M., I.D. administered the project. S.L., F.B., I.D. contributed in software. P.S., E.M., I.D. supervised the work. S.L., P.S., M.R., I.D. wrote the original draft. All authors reviewed and edited the manuscript.

## Supporting information



Supporting Information

## Data Availability

The data that support the findings of this study are available from the corresponding author upon reasonable request.
