# Supplementary material for: Hydrogel Elastic Energy: A Stressor Triggering an Adaptive Stress‐Mediated Cell Response
Source: Adv Healthc Mater. 2024 Nov 13;14(2):2402400. doi: 10.1002/adhm.202402400 (PMC11730662; doi:10.1002/adhm.202402400)
Supplement: Supplementary file 1 — Supporting Information [file ADHM-14-0-s001.pdf]

ADVANCED HEALTHCARE MATERIALS

Supporting Information

for *Adv. Healthcare Mater.*, DOI 10.1002/adhm.202402400

Hydrogel Elastic Energy: A Stressor Triggering an Adaptive Stress-Mediated Cell Response

Sara Lipari, Pasquale Sacco, Michela Cok, Francesca Scognamiglio, Maurizio Romano, Francesco Brun, Piero Giulio Giulianini, Eleonora Marsich, Finn L. Aachmann and Ivan Donati*

Supporting Information

Hydrogel elastic energy: a stressor triggering an adaptive stress-mediated cell response

Sara Lipari[a], Pasquale Sacco[a,*], Michela Cok[a], Francesca Scognamiglio[a], Maurizio Romano[a], Francesco Brun[b], Piero Giulio Giulianini[a], Eleonora Marsich[c], Finn L. Aachmann[d], and Ivan Donati[a].

[a] Department of Life Sciences, University of Trieste, Via L. Giorgieri 5, 34127 Trieste, Italy.

[b] Department of Engineering and Architecture, University of Trieste, Via A. Valerio 6/1, 34127 Trieste, Italy.

[c] Department of Medicine, Surgery and Health Sciences, University of Trieste, Piazza dell'Ospitale 1, 34129 Trieste, Italy.

[d] Norwegian Biopolymer Laboratory (NOBIPOL), Department of Biotechnology and Food Science, NTNU Norwegian University of Science and Technology, Sem Sælands vei 6/8, 7491, Trondheim, Norway

* Corresponding Author

e-mail: psacco@units.it

Figures and tables

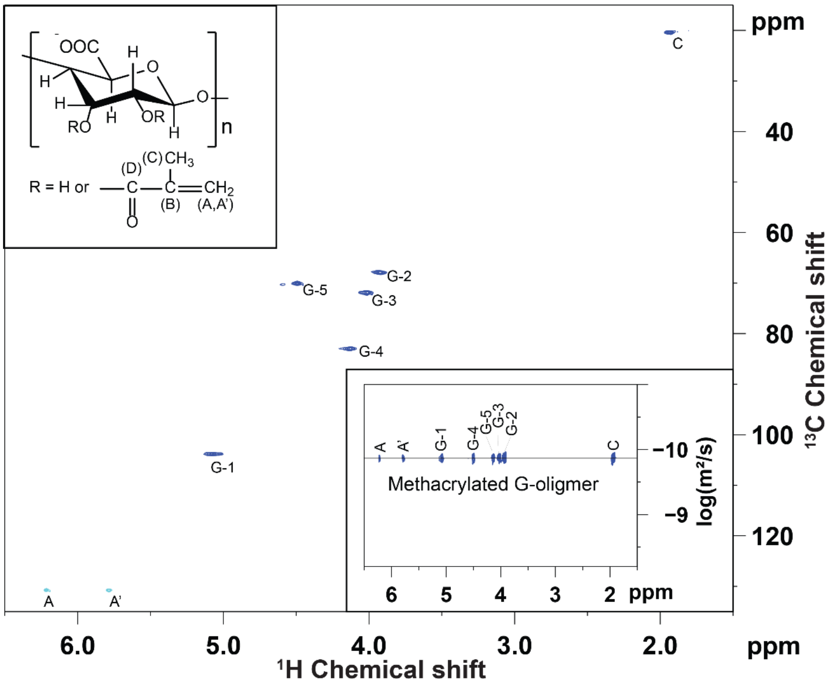

Position	$\delta_H{}^b$	$\delta_C{}^a$
methacrylate G-oligomer		
		G
1	5.06	103.8
2	3.93	68.1
3	4.01	71.9
4	4.13	83.0
5	4.49	70.1
6	-	178.0
A	6.22;5.79	130.7
B	-	138.1
C	1.94	20.5
D	-	171.2

Figure S1. **^{1}H-^{13}C HSQC and DOSY spectra of methacrylate G-oligomer in D_2O recorded at 800 Mhz and 25 °C.** The upper inlay panel shows the structure of the G-oligomer residue and the methacrylate moiety, which can be coupled to the hydroxyl group on C2 and C3. The lower inlay panel displays the DOSY spectrum, where the signals for the methacrylate moiety and the G-oligomer appear to have the same diffusion rate, indicating they are covalently bonded together. The main panel shows the ^{1}H-^{13}C HSQC spectrum with the assignment of all the peaks. The resonance assignment was carried out by starting at the anomeric signal and then following the proton-proton and proton-carbon connectivity using ^{1}H-^{1}H TOCSY, ^{1}H-^{13}C HSQC, H2BC, and HMBC. Further, the HMBC spectrum was used to identify the connectivity between the groups and their positions on the sugar ring. The methacrylate moiety was assigned in the same way. 'G-#' denotes guluronate, where '#' indicates the proton/carbon number in the guluronate sugar ring. Capital letters indicate the C/H group of the methacrylate moiety, as shown in the upper inlay panel. **Table**: Chemical shift data for methacrylate G-oligomer in D_2O recorded at 800 MHz and 25 °C. TSP (3-(Trimethylsilyl)propionate-2,2,3,3-d4) is used for chemical shift referance.'G-#' denotes guluronate, where '#' indicates the proton/carbon number in the guluronate sugar ring. Capital letters indicate the C/H group of the methacrylate moiety, as shown in the upper inlay panel.

Table S1. List of methacrylated alginates synthesized in the present work.

Sample	[Cp] (monomol$_{Alg}$/L)	$R_{M.An.}$	[NaOH] (mol/L)	Reaction time (min)	Temperature (°C)
1	0.168	5.32	0.833	60	25
2	0.168	10.6	0.833	60	25
3	0.138	5.32	0.455	60	25
4	0.168	5.32	0.833	120	25
5	0.168	5.32	2.5	60	25
6	0.253	3.55	0.833	60	25
7	0.168	5.32	0.833	20	25
8	0.168	5.32	0.25	60	25
9	0.168	5.32	0.833	60	60
10	0.168	5.32	0.833	60	25
11	0.168	15.9	0.833	60	0

Concentration of the reactants refer to the final reaction mixture. Cp is the polymer concentration expressed as monomoles of repeating units of alginate (monomol$_{Alg}$) per unit volume. $R_{M.An.}$ refers to the ratio between the moles of methacrylic anhydride ($n_{M.An.}$) and the monomoles of repeating units of alginate (monomol$_{Alg}$), ($R_{M.An.} = n_{M.An.}$/monomol$_{Alg}$).

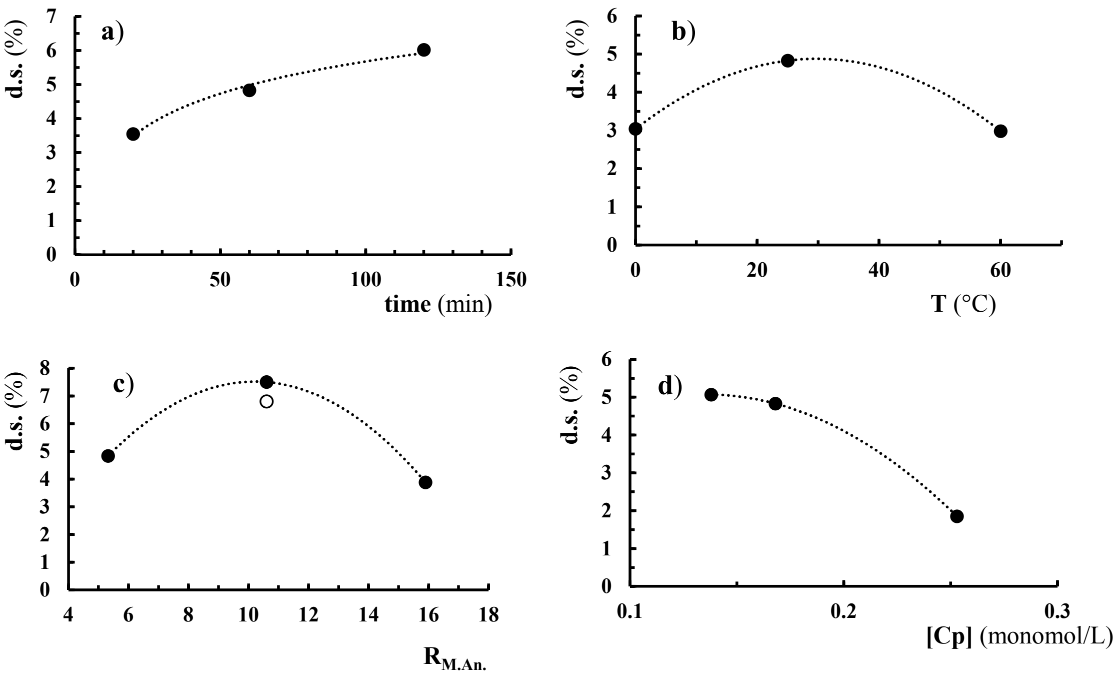

Figure S2. Dependence of the degree of substitution (d.s.) of methacrylated alginates from **a**) reaction time ([C_P] = 0.168 monomol$_{alg}$/L; $R_{M.An.}$ = 5.32; [NaOH] = 0.833 mol/L; T = 25 °C); **b**) temperature ([C_P] = 0.168 monomol$_{alg}$/L; $R_{M.An.}$ = 5.32; [NaOH] = 0.833 mol/L; reaction time = 60 min); **c**) amount of methacrylic anhydride with respect to polymer concentration ($R_{M.An.}$) ([C_P] = 0.168 monomol$_{alg}$/L; [NaOH] = 0.833 mol/L; T = 25 °C; reaction time = 60 min). The open dot represent

the degree of substitution of sample ALMA; **d**) polymer concentration (Cp) ($R_{M.An.}$ = 5.32; [NaOH] = 0.833 mol/L; T = 25 °C; reaction time = 60 min).

Table S2. Composition of the reticulating solutions.

**Reticulating condition**	**Eosin Y** (μM)	**Triethanolamine** (mM, pH = 8)	**1-vinyl-2-pyrrolidinone** (mM)
25	28	10	34
50	56	20	68
100	84	40	102

Concentrations refer to the final concentration of each agent in the solution containing ALMA.

Table S3. Different composition of the ALMA solutions. Final volume is 10 mL for all compositions.

**Sample**	**ALMA** (mg)	**10× PBS** (mL)	**Eosin Y** (0.07 M)	**1-vinyl-2-pyrrolidinone** (9.35 M)	**Triethanolamine** (0.82 M)	**H_2O**
2%50	200	1 mL	0.008 mL	0.072 mL	0.270 mL	8.65 mL
2%25	200	1 mL	0.004 mL	0.036 mL	0.135 mL	8.825 mL
1%50	100	1 mL	0.008 mL	0.072 mL	0.270 mL	8.65 mL

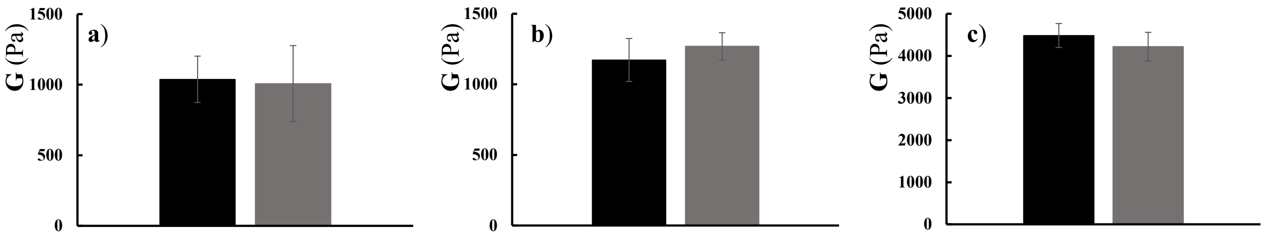

Figure S3. Shear modulus determined from the fitting of the mechanical spectra with the generalized Maxwell model (light gray) (see Appendix 2) and from the fitting of the long stress sweep data (black) (see Appendix 3) for photocrosslinked hydrogels from ALMA in condition 1%50 (**a**), 2%25 (**b**), and 2%50 (**c**) (Table S3). Values reported as average ± s.d. (n = 4). Differences among each series of data are not statistically significant.

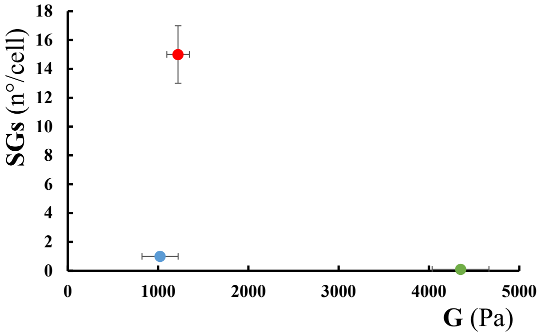

Figure S4. Dependence of the number of stress granules per cell from shear modulus for MG-63 on day 1 after hydrogel encapsulation (cell density of 3×10^6 cells/mL). Color code for hydrogels: 1%50 (blue), 2%25 (red), and 2%50 (green). See Table S3 for details.

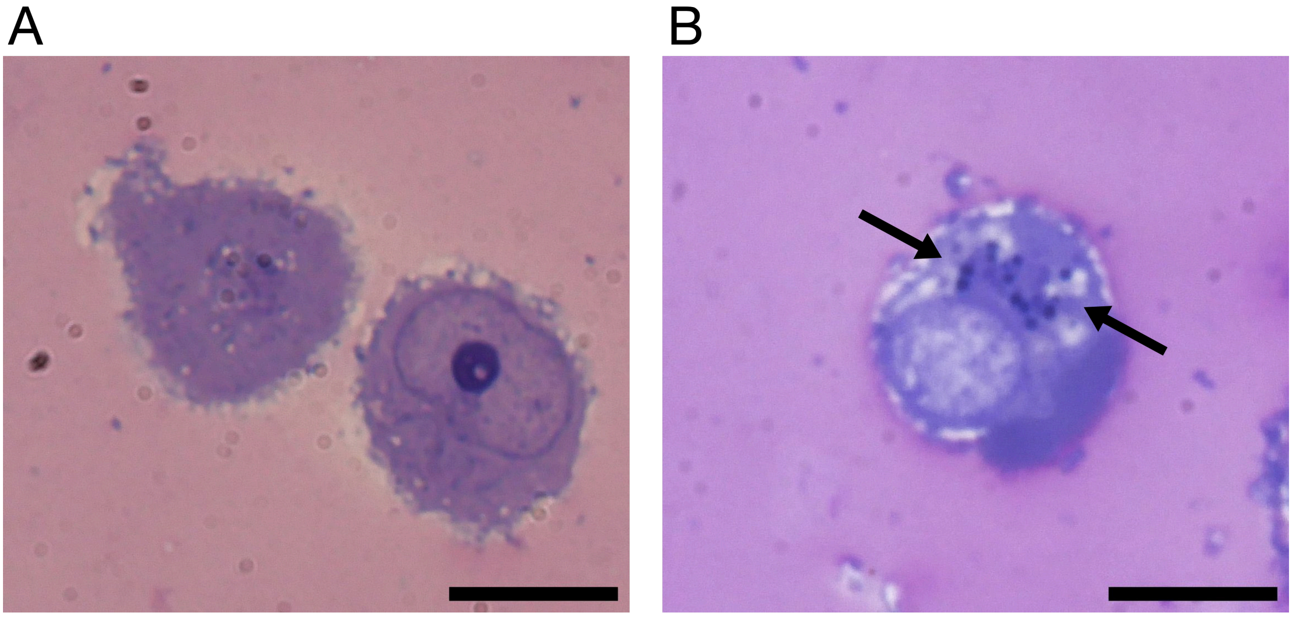

Figure S5. Light microscopy of semithin sections of 1%50 (**a**) and 2%25 (**b**) cell-laden hydrogels after 24 h of incubation in complete DMEM. Hydrogels were embedded in LR White. Scale bar 10 µm. The arrows in figure (**b**) indicate the cytoplasmic granules based on acidic components positive to toluidine blue staining.

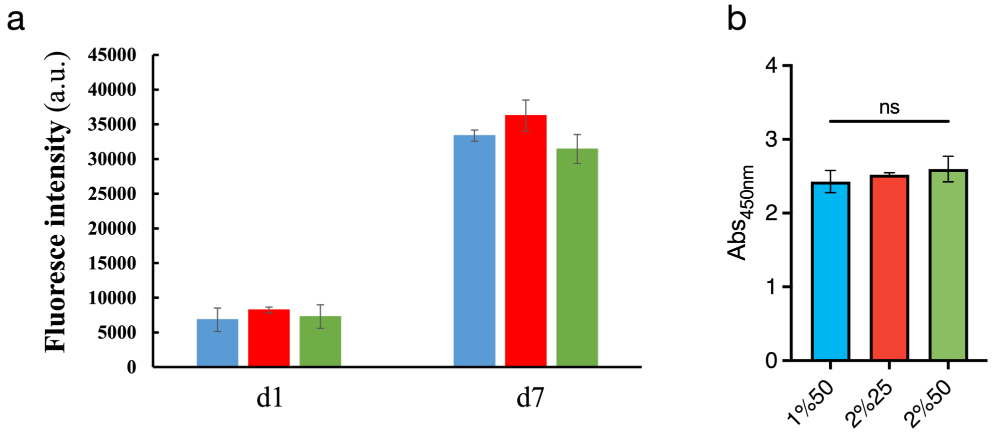

Figure S6. **a**) AlamarBlue assay for MG-63 encapsulated in the three hydrogels of Table S3 at day 1 and at day 7. Hydrogels: 1%50 (blue), 2%25 (red), and 2%50 (green). See Table S3 for details. **b**) CCK-8 assay for MG-63 encapsulated in the three hydrogels of Table S3 at day 1. See Table S3 for details.

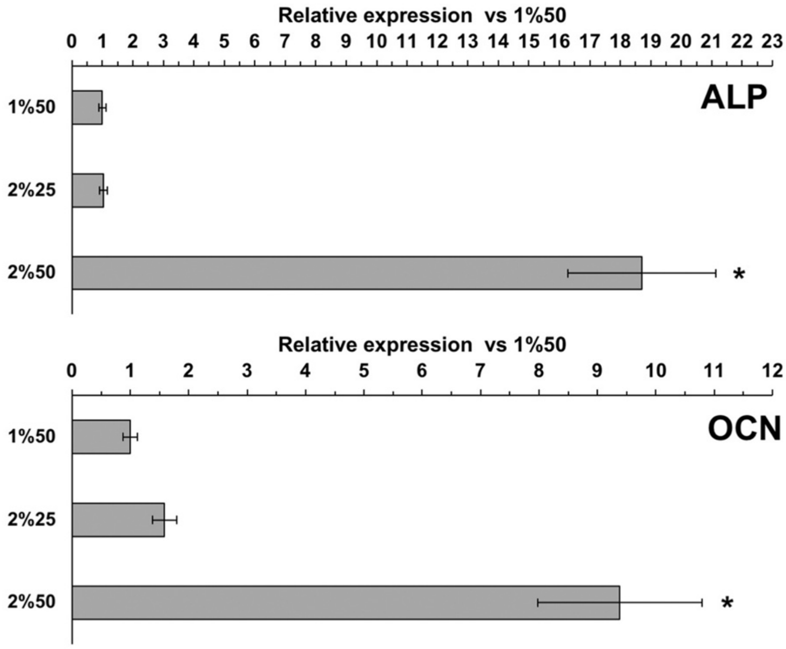

Figure S7. Expression of ALP and OCN genes in soft hydrogels. The expression of the ALP and OCN genes was calculated as described in the Materials and Methods section. The data were normalized to the housekeeping gene GAPDH, and the relative expression of both genes was calibrated to that of 1%50 (set as 1). Data are presented as mean ± s.e. (*: p-value <0.05).

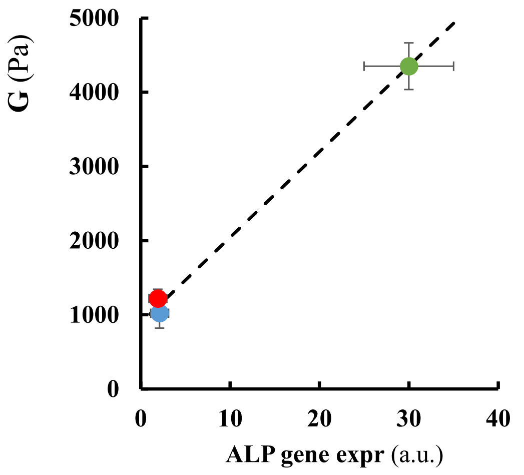

Figure S8. Correlation between shear modulus, G, and ALP gene expression for MG-63 on day 1 from encapsulation in the three hydrogels of Table S3 (density of 3 × 10^6 cells/mL). Color code for hydrogels: 1%50 (blue), 2%25 (red), and 2%50 (green).

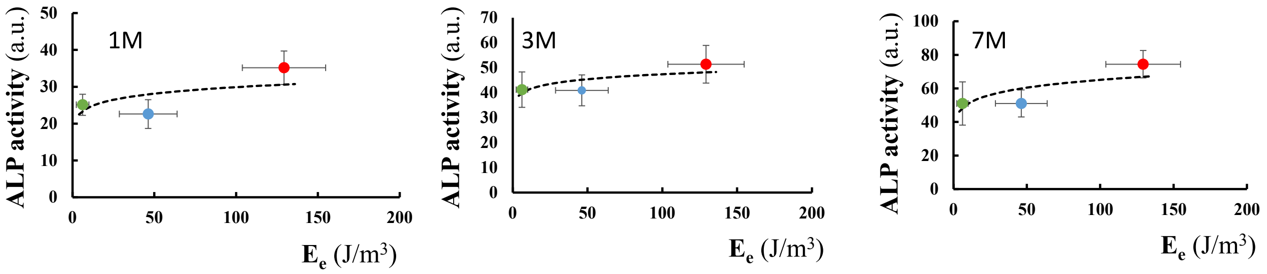

Figure S9. Correlation between ALP activity and elastic energy, E_e, for MG-63 on day 3 from encapsulation. Cell density of 1 × 10^6 cells/mL (1M), 3 × 10^6 cells/mL (3M), and 7 × 10^6 cells/mL (7M). Color code for hydrogels: 1%50 (blue), 2%25 (red), and 2%50 (green). See Table S3 for details.

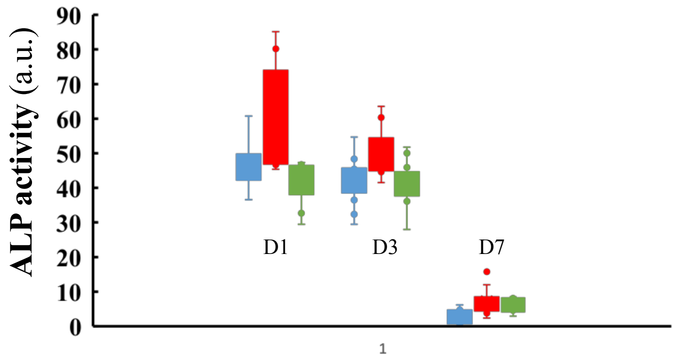

Figure S10. ALP activity of encapsulated MG-63 treated with medium without osteogenic differentiation factors at day 1, day 3 and day 7, respectively. Hydrogels: 1%50 (blue), 2%25 (red), and 2%50 (green). See Table S3 for details.

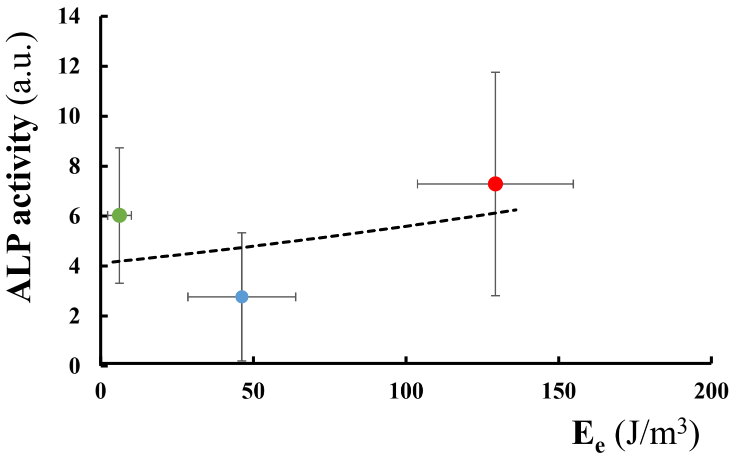

Figure S11. Correlation between ALP activity and elastic energy, E_e, for MG-63 on day 7 from encapsulation. Cell density of 3 × 10^6 cells/mL. Color code for hydrogels: 1%50 (blue), 2%25 (red), and 2%50 (green). See Table S3 for details.

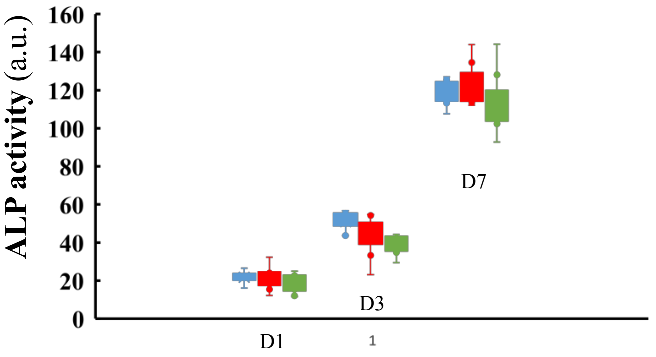

Figure S12. ALP activity of encapsulated MG-63 treated with medium supplemented with osteogenic differentiation factors at day 1, day 3 and day 7, respectively. Hydrogels: 1%50 (blue), 2%25 (red), and 2%50 (green). See Table S3 for details.

Table S4. Sequences of Real-time PCR primers**.**

Gene	Size	Forward (F) / Reverse (R)	Sequence (5' – 3')
ALP	68 bp	F	GACCCTTGACCCCCACAA
		R	GCTCGTACTGCATGTCCCCT
OCN	71 bp	F	CGAAGCCCAGCGGTGC
		R	CACTACCTCGCTGCCCTCC
GAPDH	87 bp	F	TCAAGGCTGAGAACGGGAAG
		R	CGCCCCACTTGATTTTGGAG

Appendices

Appendix 1.

Degree of substitution of methacrylated alginate as a function of the amount of methacrylic anhydride used under basic pH.

In the synthesis of methacrylated alginate under basic pH, two competing reactions take place (Scheme S1).

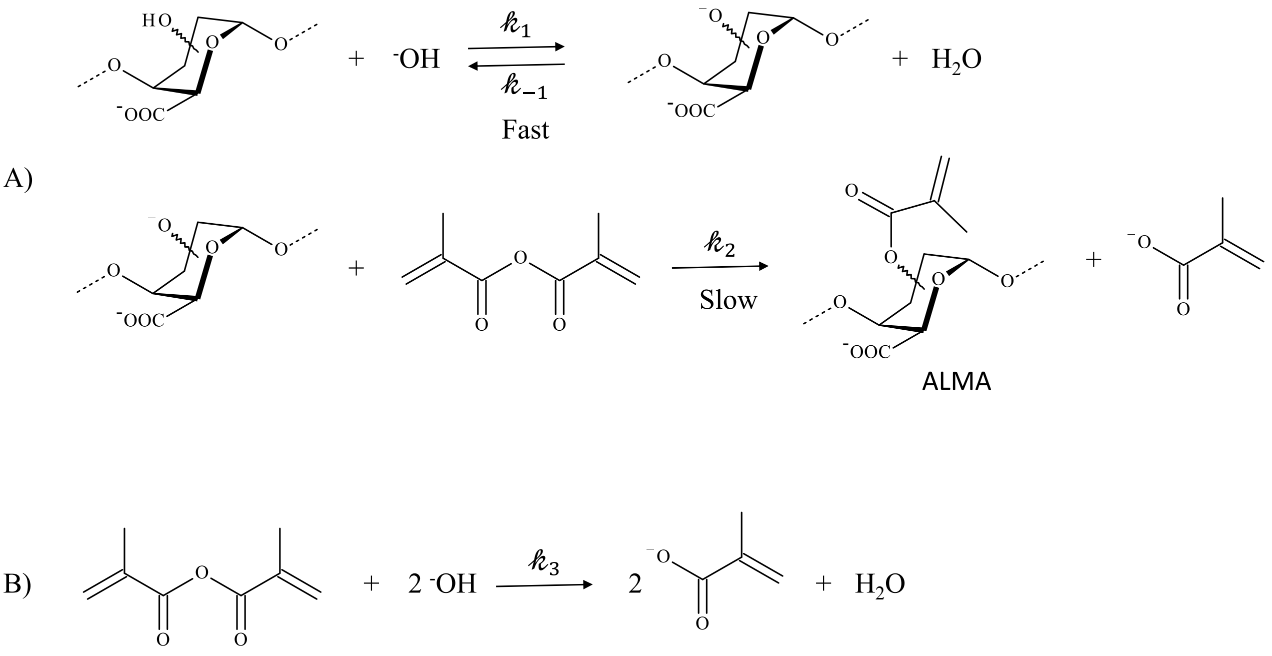

Scheme S1. Reaction scheme of the two competing reaction mechanisms involving the methacrylic anhydride.

Given the reaction scheme S1, the rate of formation of methacrylated alginate is (eq. S1):

$$v = k_2[AlgO^-][MetAnh] \qquad \text{eq S1}$$

where $[AlgO^-]$ is the concentration of alginate deprotonated at an hydroxyl group expressed as moles of repeating units per unit volume and $[MetAnh]$ is the concentration of methacrylic anhydride.
We now apply the steady state approximation to the reactive specie $AlgO^-$, *i.e.*, we assume that the rate of formation of the intermediate equals its rate of consumption (eq. S2):

$$k_1[AlgOH][OH^-] = k_{-1}[AlgO^-][H_2O] + k_2[AlgO^-][MetAnh] + k_3[OH^-]^2[MetAnh] \qquad \text{eq. S2}$$

where $[AlgOH]$ is the concentration of alginate expressed as moles of repeating units per unit volume.
Eq. S2 can be written as (eq. S3):

$$k_1[AlgOH][OH^-] = k'_{-1}[AlgO^-] + k_2[AlgO^-][MetAnh] + k_3[OH^-]^2[MetAnh] \qquad \text{eq. S3}$$

with

$$k'_{-1} = k_{-1}[H_2O]$$

We rearrange eq. S3 as follows (eq. S4):

$$k'_{-1}[AlgO^-] + k_2[AlgO^-][MetAnh] = k_1[AlgOH][OH^-] - k_3[OH^-]^2[MetAnh] \quad \text{eq. S4}$$

Eq. S4 can be further rearranged as (eq. S5):

$$[AlgO^-] = \frac{k_1[AlgOH][OH^-] - k_3[OH^-]^2[MetAnh]}{k'_{-1} + k_2[MetAnh]} \quad \text{eq. S5}$$

The rate of formation of ALMA (eq. S1) now becomes (eq. S6):

$$v = \frac{k_2k_1[AlgOH][OH^-][MetAnh] - k_2k_3[OH^-]^2[MetAnh]^2}{k'_{-1} + k_2[MetAnh]} \quad \text{eq. S6}$$

We now assume that the reaction of the deprotonated alginate with methacrylic anhydride is slow. It follows that (eq. S7):

$$k'_{-1} \gg k_2[MetAnh] \quad \text{eq. S7}$$

Eq. S6 now becomes (eq. S8):

$$v = \frac{k_2k_1[AlgOH][OH^-][MetAnh] - k_2k_3[OH^-]^2[MetAnh]^2}{k'_{-1}} = \mathcal{K}_I[AlgOH][OH^-][MetAnh] - \mathcal{K}_{II}[OH^-]^2[MetAnh]^2 \quad \text{eq. S8}$$

with $\mathcal{K}_I = \frac{k_2k_1}{k'_{-1}}$ and $\mathcal{K}_{II} = \frac{k_2k_3}{k'_{-1}}$.

We now recall that (eq. S9)

$$R_{M.An} = \frac{[MetAnh]}{[AlgOH]} \quad \text{eq. S9}$$

Eq. S8 now becomes (eq. S10):

$$v = \mathcal{K}_I[AlgOH]^2[OH^-]R_{M.An} - \mathcal{K}_{II}[AlgOH]^2[OH^-]^2{R_{M.An}}^2 \quad \text{eq. S10}$$

Equation S10 is consistent with a non-monotonic trend of the amount of ALMA from $R_{M.An.}$.

Appendix 2.

Effect of the ionic strength on alginate from *L. hyperborea* and ALMA, respectively, and determination of their stiffness parameters in unperturbed conditions.

The intrinsic viscosity, [η], was determined, for alginate from *L. hyperborea* and for ALMA, respectively, in an aqueous solution in the presence of different amounts of NaCl as supporting salt. The dependence of the intrinsic viscosity, $[\eta]_I$, from the ionic strength, I, is the following (eq. S11)[1]:

$$[\eta] = [\eta]_\infty + SI^{-1/2} \qquad \text{eq. S11}$$

where $[\eta]_\infty$ is the intrinsic viscosity at infinite ionic strength (Figure S13) and S is the slope of the curve.

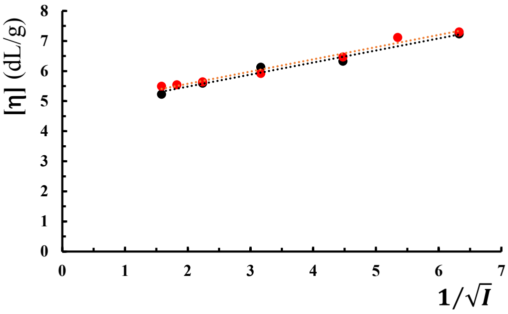

Figure S13. Dependence of the intrinsic viscosity, $[\eta]$, from the inverse of the square root of the ionic strength for alginate from *L. hyperborea* (black circles) and ALMA (red circles), respectively. Dotted lines represent the linear best fit of the experimental data (R2 > 0.98 for both cases). Each datapoint is the average of the intrinsic viscosity values obtained from Eqs. 2 and 3 ±s.e.

The slope S was used to calculate the Smidsrød's B parameter according to eq. S12[2]:

$$B = \frac{S}{([\eta]_{0.1})^{1.3}} \qquad \text{eq. S12}$$

where $[\eta]_{0.1}$ is the intrinsic viscosity measured in the presence of NaCl 0.1 M.

The determination of the intrinsic viscosity at infinite ionic strength, $[\eta]_\infty$, allowed determining the average radius of gyration, $\overline{R_{g,0}}$, in unperturbed conditions (eq. S13):

$$\overline{R_{g,0}} = \frac{1}{\sqrt{6}}\left(\frac{\bar{M}_w[\eta]_\infty}{\Phi_0}\right)^{1/3} \qquad \text{eq. S13}$$

where Φ_0 is the limiting value of the Flory viscosity constant and corresponds to $2.86 \cdot 10^{23}$ (when the intrinsic viscosity is expressed in cm^3/g). The persistence length in unperturbed conditions, q_0, can be calculated using the Doty-Benoit approach (eq. S14)[3]:

$$\left(\overline{R_{g,0}}\right)^2 = \frac{q_0 L}{3} - q_0^{\,2} + \frac{2q_0^{\,3}}{L} - \frac{2q_0^{\,4}}{L^2}\left(1 - e^{-\frac{L}{q_0}}\right) \qquad \text{eq. S14}$$

where L is the chain contour length. The virtual bond length, l, was calculated, for both unmodified alginate and ALMA, from alginate composition, assuming l = 5.17 Å and 4.35 Å for mannuronic and guluronic residues, respectively[4,5].

<u>Appendix 3</u>.

Analysis of the mechanical spectrum for photocrosslinked ALMA hydrogels using the Maxwell model.

Mechanical spectra (frequency sweep) of photocrosslinked hydrogels from ALMA are interpreted in terms of a generalized Maxwell model composed of a sequence of elements in parallel (spring and dashpot) to which an additional spring has been added. The storage, G', and loss, G", moduli are fitted as a function of the pulsation ω (= 2πν where ν is the frequency used) according to the following equations (eqs. S15 and S16)[6]:

$$G' = G_e + \sum_{i=1}^{n} \frac{G_i(\lambda_i\omega)^2}{1+(\lambda_i\omega)^2} \qquad \text{eq. S15}$$

$$G'' = \sum_{i=1}^{n} \frac{G_i\lambda_i\omega}{1+(\lambda_i\omega)^2} \qquad \text{eq. S16}$$

with

$$G_i = \frac{\eta_i}{\lambda_i}$$

where n is the number of Maxwell elements considered, G_i, η_i, and λ_i represent the spring constant, the dashpot viscosity, and the relaxation time of the i-th Maxwell element, respectively. G_e is the spring constant of the last Maxwell element which is supposed to be purely elastic. The fitting of the experimental data was performed assuming that relaxation times are scaled by a factor 10. Hence, the parameters of the fitting were G_e, η_i, and λ_1. The number of the Maxwell elements is selected to minimize the product $\chi^2 N_p$, where χ^2 is the sum of the squared errors, while $N_p(= 2 + n)$ indicates the number of fitting parameters. For all the samples analyzed, the number of Maxwell elements resulted equal to 3.

Figure S14 reports an example of a mechanical spectrum of a photocrosslinked hydrogel from ALMA and the fitting of the experimental data using the generalized Maxwell model.

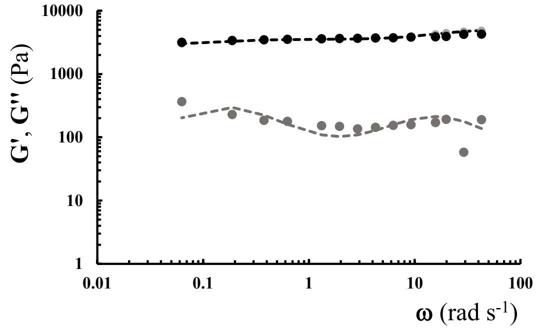

Figure S14. Dependence of storage (G', black circles) and loss (G", gray circles) moduli from pulsation for a photocrosslinked hydrogel from ALMA (2%50 sample from Table S3). Dotted lines represent the best-fit of the experimental data using eqs. S15 and S16.

The use of the generalized Maxwell model allows determining the shear modulus, G, as (eq. S17):

$$G = G_e + \sum_{i=1}^{n} G_i \qquad \text{eq. S17}$$

<u>Appendix 4</u>.

Analysis of the long stress sweep data for photocrosslinked ALMA hydrogels. Determination of shear strain and critical deformation, γ_{crit}.

Long stress sweep experiments for photoreticulated ALMA hydrogels were modeled, from the phenomenological point of view, using eq. S18.

$$\sigma = \frac{G}{1+b\gamma}\gamma \qquad \text{eq. S18}$$

Where σ is the stress, γ is the strain while *G* and *b* are fitting parameters. G corresponds to the shear modulus at $\gamma_{\rightarrow 0}$ (eq. S19).

$$G = \lim_{\gamma \to 0} \frac{d\sigma}{d\gamma} \qquad \text{eq. S19}$$

The critical strain, γ_{crit}, which marks the onset of the non-linear behavior, was defined, in line with previous works,[7,8] as (eq. S20):

$$\left.\frac{d\sigma}{d\gamma}\right|_{\gamma=\gamma_{crit}} = 0.95 \lim_{\gamma \to 0} \frac{d\sigma}{d\gamma} \qquad \text{eq. S20}$$

Eq. S20 can be rewritten as (eq. S21):

$$G|_{\gamma_{crit}} = 0.95\ G \qquad \text{eq. S21}$$

Which, combined with eq. S18, allowed determining the critical strain, γ_{crit}, as (eq. S22):

$$\frac{G}{1+b\gamma_{crit}} = 0.95G \qquad \text{eq. S22}$$

Figure S15 reported an example of a long stress sweep for a photocrosslinked ALMA hydrogel and the fitting of the experimental data using eq. S18.

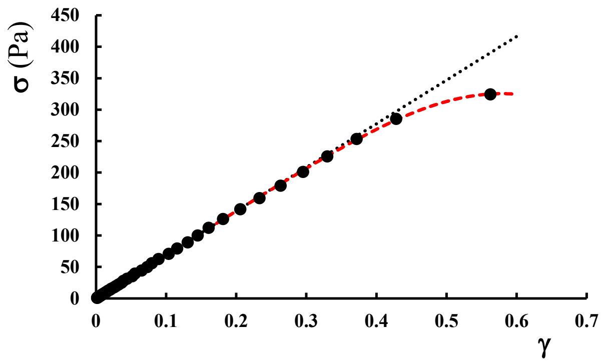

Figure S15. Experimental dependence of stress (σ) from strain (γ) for photoreticulated hydrogels from ALMA (black dots) (2%50 sample from Table S3). The black dotted curve is the linear relationship σ = G γ for all the γ range reported. The dotted red curve is the best fit of the experimental

data using eq. S18. The best-fit procedure is performed using B and G as floating parameters and minimizing the sum of the square relative error for each experimental data point.

Appendix 5.
Creep compliance analysis.

Creep compliance analyses were performed on photoreticulated hydrogels of Table S3. Figure S16 reports a typical creep compliance and recovery curve. For all samples, an immediate stress was applied to cause a deformation and the stress was maintained over time.

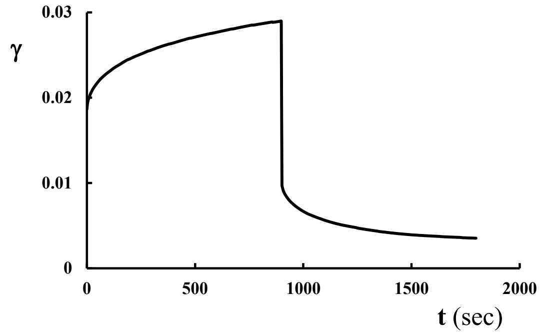

Figure S16. Example of a creep compliance and recovery curve for a photoreticulated ALMA hydrogel (2%25 in Table S3). Dependence of the deformation from time is reported.

The increase in deformation over time is analyzed in terms of the generalized Maxwell model composed of a sequence of elements in parallel (spring and dashpot) to which an additional spring has been added (eq. S23):

$$\gamma(t) = \frac{\sigma_0}{g_0 + \sum_{i=1}^{n} \frac{\lambda_i g_i}{t+\lambda_i}} \qquad \text{eq. S23}$$

where σ_0 is he initial (and constant) stress applied, λ_i and g_i represent the relaxation time and spring constant of the i-th Maxwell element, respectively. g_0 is the spring constant of the last Maxwell element which is supposed to be purely elastic. The number of Maxwell elements, n, was selected to minimize the product $\chi^2 N_p$, where χ^2 is the sum of the squared errors, while N_p indicates the number of fitting parameters.
For all the three hydrogels analyzed, the number of Maxwell elements resulted to be 2 and eq. S23 becomes (eq. S24):

$$\gamma(t) = \frac{\sigma_0}{g_0 + \frac{\lambda_1 g_1}{t+\lambda_1} + \frac{\lambda_2 g_2}{t+\lambda_2}} \qquad \text{eq. S24}$$

where 1 and 2 refer to the first and second Maxwell element, respectively (Figure S17).

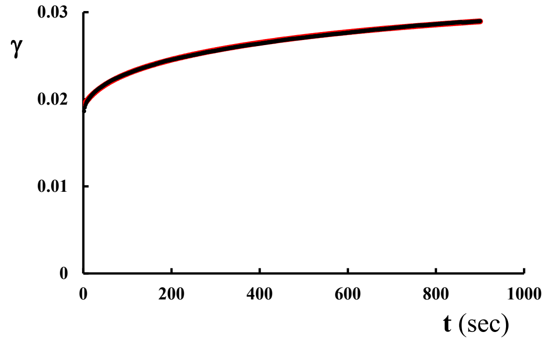

Figure S17. Experimental creep compliance (black dots) and theoretical fitting (red curve) obtained with eq. S24 (2%25 Sample in Table S3).

The shear modulus from creep compliance measurements, G_{creep}, for the three hydrogels of Table S3 was calculated as (eq. S25) and it correlates well with the values previously obtained (Figure S18):

$$G_{creep} = g_0 + g_1 + g_2 \qquad \text{eq. S25}$$

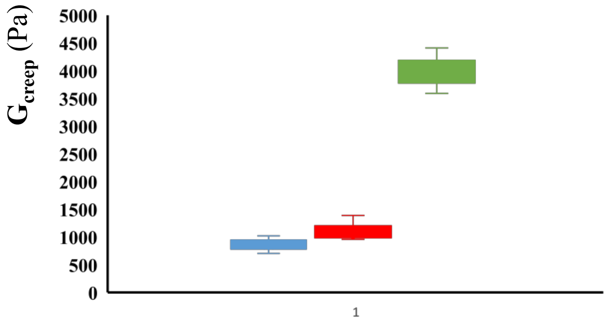

Figure S18. Shear modulus obtained from the fitting of experimental data of the creep compliance curves for the three photoreticulated hydrogels of Table S3 (eq. S15). Data are reported as mean ± s.d. (n = 3). Hydrogels: 1%50 (blue), 2%25 (red), and 2%50 (green).

The relaxation times of the first and second Maxwell elements, *i.e.*, λ_1 and λ_2, can be obtained from the fitting of the experimental datapoints using eq. S24 (Figure S19).

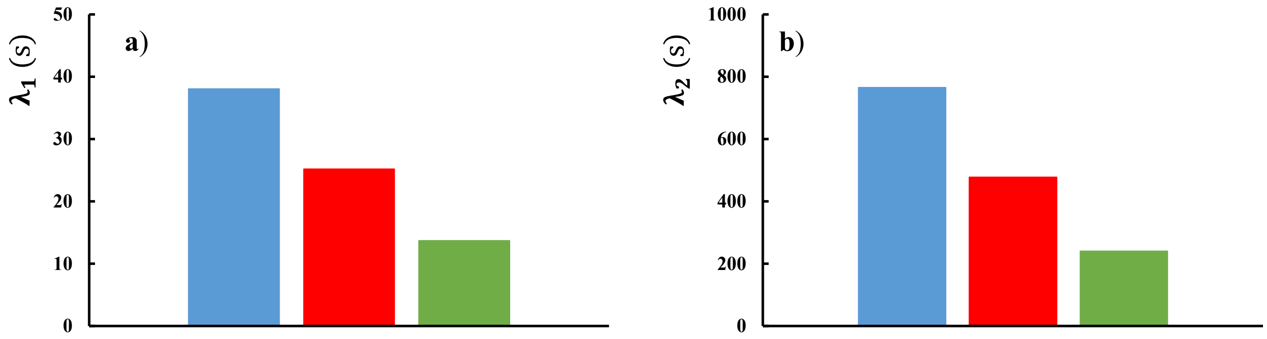

Figure S19. Relaxation time of **a**) the first (λ_1) and **b**) the second (λ_2) Maxwell element of the generalized model used for the fitting of the creep-compliance experimental datapoints. Hydrogels: 1%50 (blue), 2%25 (red), and 2%50 (green).

The viscosity of the dashpot of the first and second Maxwell elements, *i.e.*, η_1 and η_2, can be obtained from the fitting of the experimental datapoints using eq. S24 as follows (eq. S26):

$$\eta_1 = \lambda_1 g_1 \qquad \eta_2 = \lambda_2 g_2 \qquad \text{eq. S26}$$

Figure S20 reports the values of η_1 and η_2 for the three photocrosslinked hydrogels of Table S3.

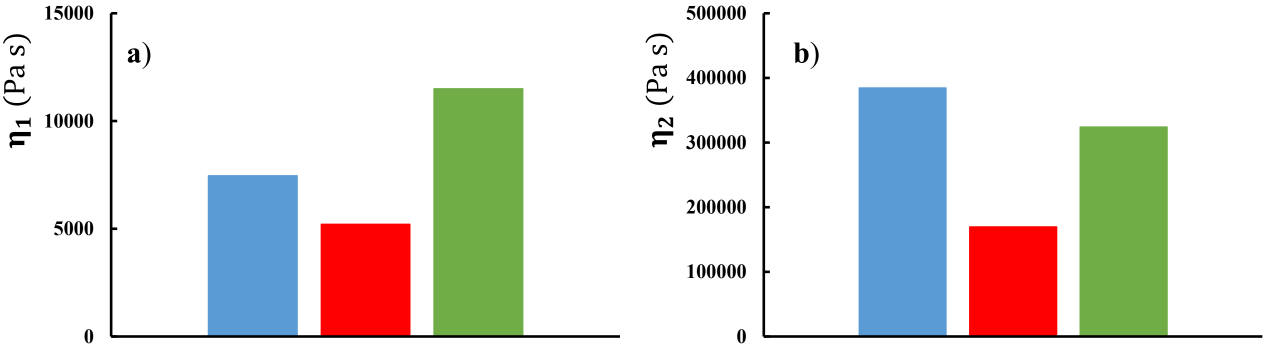

Figure S20. Viscosity of **a**) the first (η_1) and **b**) the second (η_2) Maxwell element of the generalized model used for the fitting of the creep-compliance experimental datapoints. Hydrogels: 1%50 (blue), 2%25 (red), and 2%50 (green).

The total viscosity of the photocrosslinked hydrogels of Table S3 was calculated as the sum of the two contributions from the Maxwell elements (eq. S27):

$$\eta_{tot} = \eta_1 + \eta_2 \qquad \text{eq. S27}$$

The creep time $\tau_{3/2}$ was calculated from eq. S24 calculating the time required for the strain to reach 150 % of its initial value, γ_0 (eq. S28):

$$\frac{3}{2}\gamma_0 = \frac{\sigma_0}{g_0+\frac{\lambda_1 g_1}{\tau_{3/2}+\lambda_1}+\frac{\lambda_2 g_2}{\tau_{3/2}+\lambda_2}} \qquad \text{eq. S28}$$

Values of $\tau_{3/2}$ obtained for the three hydrogels analyzed are reported in Figure S21.

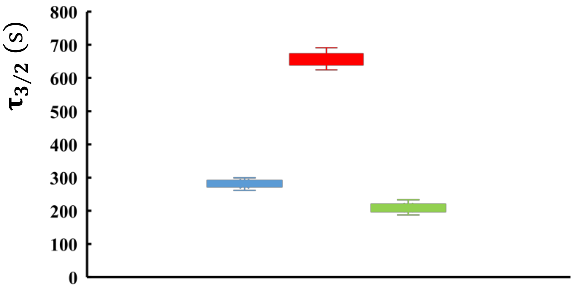

Figure S21. Creep time, $\tau_{3/2}$ determined from the creep compliance experiments according to eq. S18. Data are reported as mean ± s.d. (n = 3). Color code for hydrogels: 1%50 (blue), 2%25 (red), and 2%50 (green). See Table S3 for details.

Appendix 6.
Calculation of the average network size.

According to the rubber elasticity theory as originally proposed by Flory, the cross-linking density of the polymer network, ρ_x, is related to the shear modulus of the hydrogel (eq. S29):

$$\rho_x = \left(\frac{G}{RT}\right) \qquad \text{eq. S29}$$

where R is the universal gas constant and T in the absolute temperature. Assuming a description of the hydrogel network according to the equivalent network theory, the average mesh size is calculated (eq. S30):

$$\xi = \sqrt[3]{\frac{6RT}{\pi N_A G}} \qquad \text{eq. S30}$$

Average network size data are reported in Table S5.

Table S5. Shear modulus, G, the cross-linking density, ρ_x, and average mesh size, ξ, for the hydrogels of Table S3.

**Sample**	**G** (Pa)	$\boldsymbol{\rho_x}$ (mol/m^3)	$\boldsymbol{\xi}$ (nm)
1%50	862.6 ± 158.6	0.38 ± 0.07	20.3 ± 1.2
2%25	1120.8 ± 234.7	0.50 ± 0.10	18.6 ± 1.3
2%50	3987.0 ± 410.3	1.76 ± 0.18	12.3 ± 4.2

References

1. Smidsrød, O. & Haug, A. Estimation of the relative stiffness of the molecular chain in polyelectrolytes from measurements of viscosity at different ionic strengths. *Biopolymers* **10**, 1213–1227 (1971).

2. Smidsrød, O. & Haug, A. Estimation of the relative stiffness of the molecular chain in polyelectrolytes from measurements of viscosity at different ionic strengths. *Biopolymers* **10**, 1213–1227 (1971).

3. Cok, M. *et al.* N-isopropyl chitosan. A pH- and thermo-responsive polysaccharide for gel formation. *Carbohydrate Polymers* **230**, 115641 (2020).

4. Donati, I., Asaro, F. & Paoletti, S. Experimental evidence of counterion affinity in alginates: The case of nongelling ion Mg^{2+}. *Journal of Physical Chemistry B* **113**, 12877–12886 (2009).

5. Donati, I., Cesàro, A. & Paoletti, S. Specific interactions versus counterion condensation. 1. Nongelling ions/polyuronate systems. *Biomacromolecules* **7**, 281–287 (2006).

6. Turco, G. *et al.* Mechanical spectroscopy and relaxometry on alginate hydrogels: A comparative analysis for structural characterization and network mesh size determination. *Biomacromolecules* **12**, 1272–1282 (2011).

7. Marsich, E. *et al.* Polysaccharide-based polyanion-polycation-polyanion ternary systems in the concentrated regime and hydrogel form. *Macromolecular Chemistry and Physics* **214**, 1309–1320 (2013).

8. Sacco, P., Cok, M., Asaro, F., Paoletti, S. & Donati, I. The role played by the molecular weight and acetylation degree in modulating the stiffness and elasticity of chitosan gels. *Carbohydrate Polymers* **196**, 405–413 (2018).
